# Quantum control in size selected semiconductor quantum dot thin films

**DOI:** 10.1515/nanoph-2024-0529

**Published:** 2025-01-16

**Authors:** Victor Kärcher, Tobias Reiker, Pedro F.G.M. da Costa, Andrea S.S. de Camargo, Helmut Zacharias

**Affiliations:** Center for Soft Nanoscience, University of Münster, 48149 Münster, Germany; São Carlos Institute of Physics, University of São Paulo, São Carlos, SP 13566-590, Brazil; Federal Institute for Materials Research and Testing (BAM), 12489 Berlin, Germany; Friedrich-Schiller University Jena (FSU), 07743 Jena, Germany

**Keywords:** nonlinear nanophotonics, quantum interference, third harmonic, coherent control, thin films, quantum dots

## Abstract

We introduce a novel technique for coherent control that employs resonant internally generated fields in CdTe quantum dot (QD) thin films at the *L*-point. The bulk band gap of CdTe at the *L*-point amounts to 3.6 eV, with the transition marked by strong Coulomb coupling. Third harmonic generation (*λ*_3_ = 343 nm, *hν* = 3.61 eV) for a fundamental wavelength of *λ*_1_ = 1,030 nm is used to control quantum interference of three-photon resonant paths between the valence and conduction bands. Different thicknesses of the CdTe QDs are used to manipulate the phase relationship between the external fundamental and the internally generated third harmonic, resulting in either suppression or strong enhancement of the resonant third harmonic, while the nonresonant components remain nearly constant. This development could pave the way for new quantum interference–based applications in ultrafast switching of nanophotonic devices.

## 1 Introduction

Control and manipulation of opto-electronic properties of quantum systems is one of the main goals in many areas of physics [1], [2] and chemistry [3], [4]. In recent years, significant advances in nanofabrication [5], [6] have led to remarkable improvements in the control of electric charges, which provide excellent conditions for the use of nanotechnologies in quantum information [7] and quantum communication [8]. Especially quantum dots (QDs) are promising candidates for quantum (nano) devices not only due to their unique electrical and optical properties [9] but also their easy implementation in nanophotonic devices [10], [11]. Their size and composition can be precisely controlled during synthesis allowing for a controlled design of their band structure and energy levels [9]. Their tunability [12] enables the manipulation of inter- and intraband processes [13] making semiconductor QDs beneficial for various applications [14]. Quantum dot (QD) thin films are particularly promising for enabling direct coupling between different emitters in ensemble setups [15] and for achieving precise charge carrier control on a femtosecond timescale [16].

Precisely controlling parameters in physical [17], [18] and chemical [3], [19], [20], [21] processes on a femtosecond time scale is a highly sought-after goal in many scientific disciplines. This process is known as coherent control. The idea behind coherent control was originally proposed by Manykin and Afanasev [22]. A process was predicted in which the charge transfer between two states can be optically controlled when tuning the driving field into resonance of the atomic system. This was first demonstrated experimentally by Aron and Johnson [23] in a three-photon resonant system. Quantum interference of different paths connecting the initial and final states led to the absence of the multiphoton ionization signal, which is directly connected to third harmonic generation [24]. Several studies showed the suppression effects in even [22], [25], [26], [27], [28] and in odd [24], [29], [30], [31], [32], [33], [34], [35] photon resonant system. The interference that cancels two-photon resonant systems is induced by two phase-shifted Rabi oscillations, which originate from the external laser source and internally generated fields due to parametric four-wave mixing where the phase mismatch Δ*k* of the fields is zero [27]. In odd parity systems, the phase mismatch Δ*k* between the fundamental and the third harmonic leads to strong absorption of the material around resonance, and only constant phase relation with stimulated emission is possible. Free wave propagation with random phase relation is suppressed [31], a prerequisite for the medium to become transparent to the internally generated wave. Achieving this transparency requires a large phase mismatch, which is unattainable in gaseous media due to their refractive index being only marginally greater than one. Due to inversion symmetry, the internally generated field is 180° out of phase to the driving field, and thus the material depolarizes. Once the magnitude for total destructive interference is achieved, quantum interference prevents further harmonic generation in atoms. Thus, coherent control is typically achieved using two external fields to manipulate quantum transitions [17], [18], [36], [37], [38]. By utilizing internally generated fields in semiconductor QD thin films, which exhibit significant differences in refractive index leading to a large phase mismatch between the fundamental and third harmonic, the resonant one-photon absorption of the medium can be coherently controlled. This control renders the QDs transparent to the resonantly generated wave at a specific film thickness. Quantum control is thus achieved using only a single external field, simplifying the experimental setup. Thin QD films are particularly well-suited for this approach, providing a robust platform for ultrafast optical applications, such as nonlinear signal modulation and the coupling of emitters in ensemble configurations [15]. Additionally, QDs and nanoparticles offer size-dependent resonant and nonresonant characteristics [39], introducing experimental opportunities like tunability not typically accessible with gaseous media.

In this work, we present a novel method for coherent control of an injected current, which will be modulated by internally generated fields in a three-photon resonant system. The phase mismatch between the third harmonic and the fundamental beam in CdTe QDs is used to modulate the third-order nonlinear and size-dependent susceptibility at the *L* point of the Brillouin zone ( $\Delta E_{g}^{L}=3.6$ eV) and thus enabling coherent control of the injected current by the film thicknesses. By employing high-intensity pulses, the phase mismatch is modulated and controlled by the number of electrons in the conduction band, which opens up new opportunities for their use in nanophotonic devices.

## 2 Quantum interference

In this section, we briefly show the connection between the transition rate and the third order nonlinear susceptibility tensor *χ* ^(3)^(−3*ω*, *ω*, *ω*, *ω*). A detailed calculation can be found in the Supplementary Material. The behavior of a particle in an external field can be described by the semi-classical interaction Hamiltonian *H*_int_ = −*e*/(*mc*)*pA*(*t*), where *e* denotes the electric charge, *m* the electron mass, *c* the light velocity, *p* the momentum operator, and *A*(*t*) the time dependent external field. By solving the time-dependent Schrödinger equation *H*_int_|*ψ*⟩ = *iℏ∂*_*t*_|*ψ*⟩, the transition amplitudes *c*_*m*_(*t*) can be calculated when the wavefunction is expanded as a superposition of the basis set |*m*⟩

(1) $$|\psi \left(t\right)〉=\underset{m}{\sum }c_{m}\left(t\right)\exp\left(-i\omega_{m}t\right)|m〉,$$

where |*c*_*m*_(*t*)|^2^ is the probability of finding a particle in state |*m*⟩ at time *t*. When time-dependent perturbation theory is applied, the coefficient *c*_*m*_(*t*) can be expanded on the power dependence (*q*), where *q* determines the number of photons with frequency *ω*_*m*_ involved in the transition. In third harmonic generation, the superposition of the driving external and generated harmonic internal field can be written as

(2) $$\begin{array}{ll} A\left(z,t\right) & =A_{\omega }\exp\left(-\left(\omega +i\Gamma \right)t+kz\right)\\ & \;+A_{\omega }^{*}\exp\left(\left(\omega -i\Gamma \right)t-kz\right)\\ & \;+A_{\omega_{3}}\exp\left(-\left(\omega_{3}+3i\Gamma \right)t+k_{3}z\right)\\ & \;+A_{\omega_{3}}^{*}\exp\left(\left(\omega_{3}-3i\Gamma \right)t-k_{3}z\right), \end{array}$$

where *ω* = *ω*_1_ and *k* = *k*_1_ describe the fundamental frequency and wave number, *ω*_3_ and *k*_3_ the third harmonic frequency and wave number, and Γ the linewidth. Now we can calculate the transition amplitude $c_{m}^{\left(q\right)}\left(t\right)$ for one photon from the initial state *i* to the final state *f* at position *z* induced by the frequency *ω*_3_ by integrating the Schrödinger equation once

$$c_{f}^{\left(1\right)}\left(z,t\right)=-\frac{e}{\hbar cm}p_{fi}A_{\omega_{3}}\frac{{\text{e}}^{-\text{i}\left(\omega_{3}-\omega_{fi}+i\Gamma \right)t}}{\omega_{3}-\omega_{fi}+i\Gamma }e^{ik_{3}z},$$

with

(3) $$p_{fi}=〈f|p|i〉$$

and the three-photon transition amplitude for *ω* by integrating the Schrödinger equation three times

(4) $$\begin{array}{ll} c_{f}^{\left(3\right)}\left(z,t\right) & =-{\left(\frac{e}{\hbar cm}\right)}^{3}\underset{f,l,j,i}{\sum }\frac{p_{fl}A_{\omega }p_{lj}A_{\omega }p_{ji}A_{\omega }}{\left(\omega -\omega_{ji}\right)\left(2\omega -\omega_{li}\right)}\\ & \;\times \frac{{\text{e}}^{-\text{i}\left(3\omega -\omega_{fi}+3i\Gamma \right)t}}{\left(3\omega -\omega_{fi}+3i\Gamma \right)}e^{3i}kz. \end{array}$$

By calculating the transition rate $T=\frac{d}{dt}|c_{f}^{\left(1\right)}\left(z,t\right)+c_{f}^{\left(3\right)}\left(z,t\right){|}^{2}$ , it is obvious that interference between the one photon and three photon process occurs. Note, that the first two transition probabilities become zero in centrosymmetric solid state systems when summing over the whole Brillouin zone, since *p*_*fi*_(*k*) = −*p*_*fi*_(−*k*) drives the fields out of phase by a factor of *π*. Only in noncentrosymmetric problems, these two transitions will be nonzero. By using the *p* = *imωx* and the definition of the third order susceptibility tensor

(5) $$\begin{array}{ll} & \chi^{\left(3\right)}\left(-3\omega ,\omega ,\omega ,\omega \right)\\ & \;=\frac{N}{\epsilon_{0}}\frac{e^{4}}{3!\hbar^{3}}\underset{f,l,j,i}{\sum }\frac{x_{if}x_{fl}x_{lj}x_{ji}}{\left(\omega -\omega_{ji}\right)\left(2\omega -\omega_{li}\right)\left(3\omega -\omega_{fi}-3i\Gamma \right)}, \end{array}$$

where *N* is the number of oscillators per unit, volume, *ϵ*_0_ is the permeability, the interference term $c_{f}^{\left(1\right)}\left(z,t\right){\left(c_{f}^{\left(3\right)}\left(z,t\right)\right)}^{*}$ can be written as

(6) $$\begin{array}{ll} & \frac{d}{dt}c_{f}^{\left(1\right)}\left(z,t\right){\left(c_{f}^{\left(3\right)}\left(z,t\right)\right)}^{*}{|}_{t=0}\\ & \;={\left(\frac{N}{\epsilon_{0}}\frac{e^{4}}{3!\hbar^{3}}\right)}^{-1}{\left(\frac{e}{\hbar mc}\right)}^{4}{\left(m\omega \right)}^{4}\chi^{\left(3\right)}\\ & \;\;\times \left(-3\omega ,\omega ,\omega ,\omega \right)\times A_{3\omega }A_{\omega }^{3}i{\text{e}}^{\text{i}\Delta kz}\delta \left(\omega_{fi}-\omega_{3}\right) \end{array}$$

with Δ*k* = *k*_3_ − 3*k*. Note that this interference term only remains when *ω*_3_ = *ω*_*fi*_. Thus, we can define a $\chi_{\text{eff}}^{\left(3\right)}\left(-3\omega ,\omega ,\omega ,\omega \right)$ with

(7) $$R\left(\chi_{\text{eff}}^{\left(3\right)}\left(-3\omega ,\omega ,\omega ,\omega \right)\right)=\chi^{\left(3\right)}\left(-3\omega ,\omega ,\omega ,\omega \right)\sin\left(\Delta kz\right)$$

and

(8) $$I\left(\chi_{\text{eff}}^{\left(3\right)}\left(-3\omega ,\omega ,\omega ,\omega \right)\right)=\chi^{\left(3\right)}\left(-3\omega ,\omega ,\omega ,\omega \right)\cos\left(\Delta kz\right).$$

The gain of the resonant harmonic is determined by the real part of the transition amplitude, as described by eq. (7), while the absorption of the resonant harmonic corresponds to the imaginary part, as given by eq. (8). This establishes a direct connection between absorption and gain. At *z* = 0, the modulation of the gain for the resonant harmonic is zero, whereas the modulation of the absorption is maximal. As the propagation distance *z* in the resonant medium increases, the absorption decreases, eventually rendering the medium transparent to the resonant third harmonic. This transparency manifests as an increase in the measured signal of the resonant third harmonic. At this stage, the gain is governed solely by the real part of the transition amplitude described in eq. (7), and the gain as a function of sample thickness *z* is expressed as:

(9) $$E\left(\omega_{3},z,t\right)\propto \chi_{\text{eff}}^{\left(3\right)}\left(-3\omega ,\omega ,\omega ,\omega \right)E^{3}\left(\omega ,z,t\right)\sin\left(\Delta kz\right),$$

where *E*(*ω*_3_, *z*, *t*) is the electric field of the resonant frequency *ω*_3_, modulated by sin(Δ*kz*). This equation is only nonzero when there is a nonzero phase mismatch, Δ*k* ≠ 0, between the driving field and the internally generated third harmonic. Under perfect phase-matching conditions (Δ*k* = 0), the modulation of the resonant field *E*(*ω*_3_, *z*, *t*) vanishes, and absorption dominates the process. Therefore, a nonzero phase mismatch is essential for achieving coherent control of transition amplitudes in odd-photon resonant systems.

At the sample’s initial interface, the internally generated field is suppressed. As the propagation distance *z* increases, the phase mismatch between the fundamental and third harmonic fields leads to a modulation of the propagated third harmonic field. This propagation distance *z* can be experimentally controlled by varying the thickness of the CdTe QD coating. A detailed derivation of the propagated and stimulated fields, based on Maxwell’s equations with a nonlinear source term, is provided in the Supplementary Information (eqs. (S31)–(S34)).

## 3 Methods and materials

### 3.1 Bulk cadmium telluride

**Figure 1:**
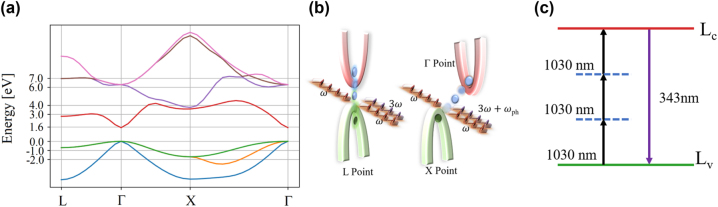
Energy levels of Bulk CdTe. (a) Band structure of bulk CdTe along the Brillouin zone calculated with octopus [40], [41], [42], [43]. The direct band gap at the *L* point and the indirect band gap from the Γ point to the *X* point correspond to the photon energy of the third harmonic with a wavelength of *λ*_3_ = 343 nm for a fundamental wavelength of *λ*_1_ = 1,030 nm. (b) Schematic drawing of the direct band gap at the *L* point and the indirect band gap between the Γ point and the *X* point. For this indirect transition, a phonon with frequency *ω*_*ph*_ is required. In (c), the resonant three-photon process between the valence band *L*_*v*_ and the first conduction band *L*_*c*_ at the *L* point is depicted. This resonant transition is necessary for quantum interference.

CdTe is a type II–VI semiconductor with a zinc blende structure (space group *F* $\bar{4}$ 3*m*). Figure 1(a) shows the calculated band structure of bulk CdTe along the $\bar{\Gamma L}$ and $\bar{\Gamma X}$ directions [40], [41], [42], [43] (see Supplementary Information). The direct band gap at the Γ point is $E_{g}^{\Gamma }=1.6$ eV, at the *L* point $E_{g}^{L}=3.6$ eV, and at the *X* point $E_{g}^{X}=5.6$ eV. The wavelength of the third harmonic is 343 nm (3.61 eV), and thus a resonant behavior at the *L* point is expected. Several studies provide evidence for strong excitonic behavior at the *L* point [44], [45], [46], [47], thus, suggesting strong one-photon absorption in this region. This is crucial to achieve an optically thick medium in order to suppress the random phase relation between a free traveling generated wave and the fundamental wave [30], [31]. The *L* point of CdTe corresponds to an inflection point in the valence and conduction band separation where electron and hole have equal phase velocities [44], [46], [47]. In general, Coulomb effects arise in any saddle-type critical points where electron–hole correlations are strong [46], leading to significant dipole-coupled transitions. These transitions are essential for generating stimulated resonant harmonic fields with a constant phase relation to the seed pulse [30], [31], from which oscillating absorption and transmission characteristics emerge [22].

Besides the resonant behavior of the third harmonic, the interaction between electrons/excitons with phonons induces a coupling of the indirect band gap between the Γ and *X* points, which also amounts to $E_{g}^{\Gamma -X}=3.6$ eV as can be seen from Figure 1(a) and (b) giving rise to blue shifted harmonics. Table S1 provides the phonon energies of bulk CdTe [48]. Note, that the phonon energies remain the same when considering the QD counterpart, while the exciton–phonon coupling strongly depends on the QD size and is different from the coupling in bulk material, a phenomenon which is known as phonon bottleneck [49]. When going from bulk to QD, the coupling first reduces until some critical size and increases afterward with decreasing size. For CdTe, this critical size is at about *d* = 4 nm [50], [51].

### 3.2 CdTe quantum dots

The synthesis of CdTe QDs was performed based on the procedure reported in Ref. [12], [52], [53], [54]. First, 30 mL of ultra-pure water was added to a three-necked flask under magnetic stirring and argon flow to remove dissolved oxygen. Then, 17.13 mg of cadmium chloride (CdCl_2_·2.5H_2_O) and 10 µL of 3-mercaptopropionic acid (MPA) were added, and the pH was adjusted to pH 8 using a 0.5 M NaOH solution. In a second three-neck flask under a dry argon atmosphere, 7.97 mg of metallic tellurium (Te^0^) and 12.53 mg of sodium borohydride (NaBH_4_) were added. Then, 3.12 mL of argon-saturated ultra-pure water was slowly mixed, and the solution was slowly heated to 70 °C. Around this temperature, the solution started to turn pink. When the remaining metallic tellurium was reduced to NaHTe, 2.50 mL of the light pink solution was quickly added to the first flask using a syringe. The resulting solution immediately turned light yellow, indicating the formation of CdTe seeds. The ice bath was removed, and heating to 90 °C was started. Upon reaching this temperature, eight samples were collected for measurements after the following reaction times of 0.5, 1.0, 2.0, 4.0, 6.0, 24, and 48 h. The resulting quantum dot have sizes between 2 and 4 nm and band gaps between 2.54 eV and 1.77 eV at the Γ point. The band gap of the solid is *E*_*g*,*s*_ = 1.6 eV. However, quantum confinement does not occur at the *L* and *X* points, as observed in the absorption spectrum of the QDs shown in Figure S3 in the Supplementary Material. Consequently, the three-photon resonant behavior at the *L* point persists across all sizes, leading to the expectation of quantum interference.

**Figure 2:**
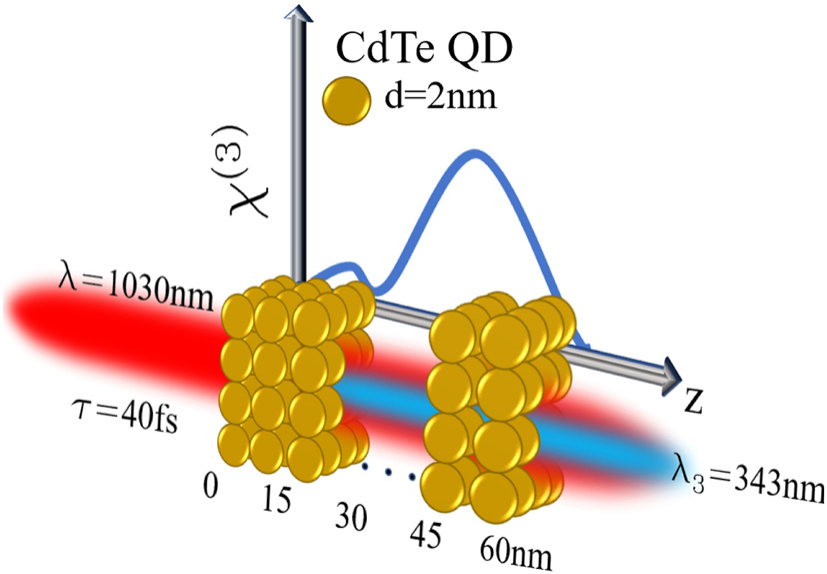
Schematic representation of the experiment. In order to coherently control the resonant nonlinear susceptibility, different CdTe QD film thicknesses are coated onto a borosilicate substrate.

The CdTe QDs have been deposited on a borosilicate glass wafer, with quantum interference controlled by varying the thickness *z* of the film in the range from 10 to 60 nm. Figure 2 illustrates the schematic setup of the new method designed to coherently control the resonant nonlinear susceptibility *χ* ^(3)^ of the CdTe QDs at the *L* point, as described by eq. (7). Thus, third harmonic generation is employed to detect quantum interference. Using femtosecond pulses (40 fs, up to 80 µJ, 50 kHz repetition rate) from a fiber laser (Active Fiber Systems) with peak intensities of up to 5 × 10^13^ W/cm^2^ are utilized, with a central wavelength of *λ* = 1,030 nm, to produce the resonant third harmonic at *λ*_3_ = 343 nm (see Section 6 in the Supplementary Material).

## 4 Results and discussion

### 4.1 Third harmonic in CdTe quantum dots

**Figure 3:**
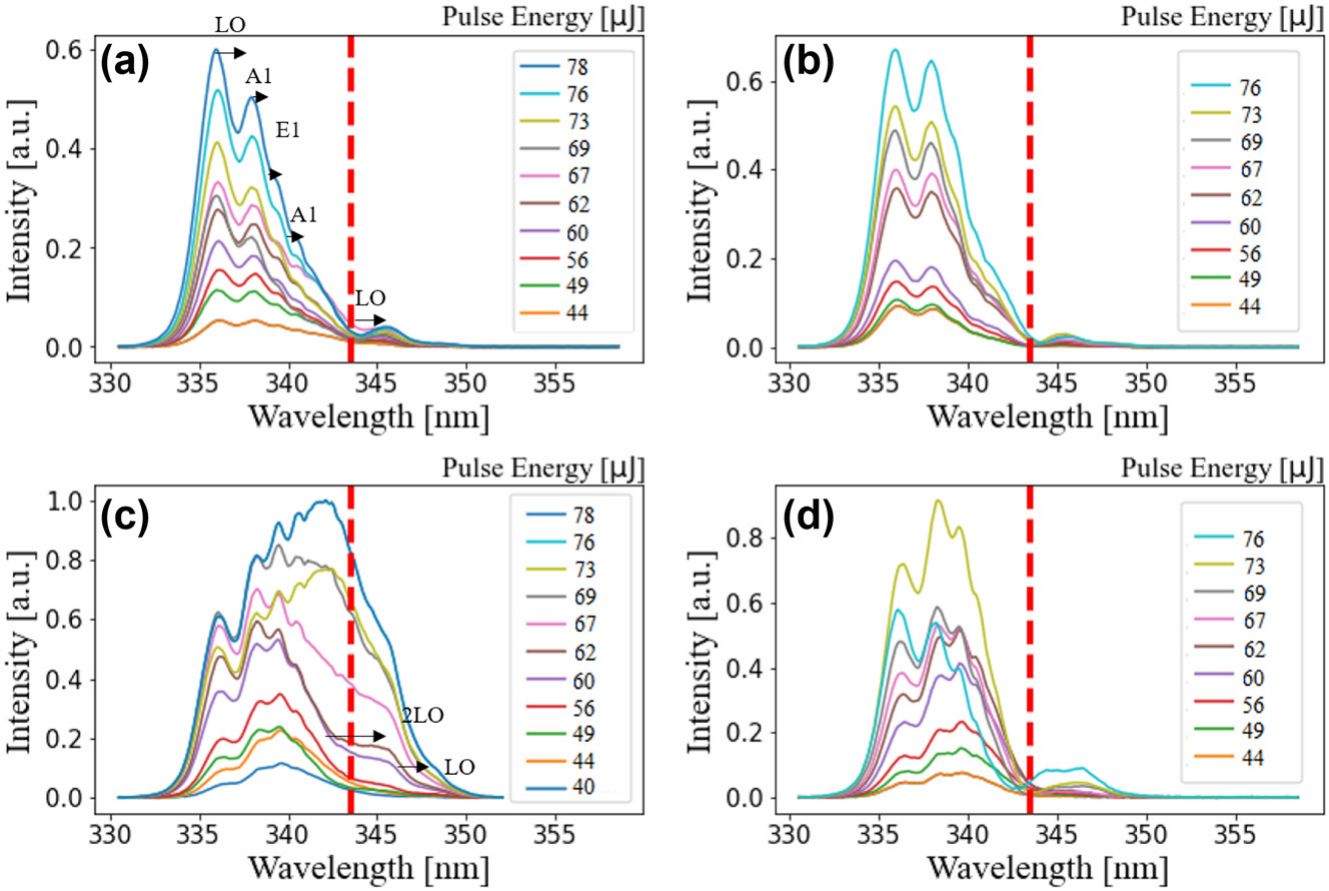
Generated third harmonic yield for CdTe QDs with a size of 1.94 nm and for thicknesses of (a) 10 nm, (b) 27 nm, (c) 45 nm, and (d) 60 nm. Spectral broadening is observed in (c), while in (d), the coating reached a critical thickness and the QDs are destroyed at pulse energies of 76 μJ. The yield is normalized to the highest peak in (c). This will be the normalization constant for all sizes and films. In addition, the shift of the peaks according to the LO and TO modes *A*_1_ and *E*_1_ of CdTe is indicated.

This section outlines the spectral characteristics of the third harmonic generated using CdTe quantum dots. Figure 3(a)–(d) presents the third harmonic yield for four different film thicknesses, with CdTe QD sizes of *d* = 1.94 nm, as detailed in Table 1. The fundamental laser power is used as the variable parameter to determine the nonlinear order of the process, which is essential for confirming a *χ* ^(3)^ process that drives the modulation of the quantum interference effect. All graphs are normalized to the maximum yield observed in Figure 3(c).

**Table 1:** Characteristics of the CdTe quantum dots used in this work. The first column displays the reaction time; the second provides the band gap *E*_*g*_ at the Γ point (luminescence emission maximum). The third column shows the diameter derived with the absorption method (ABS), and the fourth shows the diameter measured with dynamic light scattering (DLS).

QD	*E*_*g*_ [eV]	*d* [nm] (ABS)	*d* [nm] (DLS)
0.5 h	2.54	1.94	–
1 h	2.47	2.01	2.9
2 h	2.41	2.08	3.03
4 h	2.34	2.16	3.03
6 h	2.25	2.30	3.33
24 h	1.97	2.95	3.94
48 h	1.77	4.04	–

For a coated thickness of 10 nm, as shown in Figure 3(a), the resonant harmonic (marked by a red line) is suppressed, while the nonresonant components are amplified. A similar trend is observed for a coated thickness of 27 nm in Figure 3(b), where no significant changes occur compared to Figure 3(a), aside from a slight increase in signal strength. Additionally, new peaks emerge in the spectrum, with spacing that appears to correspond to the phonon lines of bulk CdTe. The transverse modes *A*_1_ and *E*_1_ are observed at 123 cm^−1^ and 142 cm^−1^, respectively, while the LO mode of the bulk crystal appears at 166 cm^−1^ and the second LO at 333 cm^−1^, as indicated in Figure 3(a) and (c). This off-resonant signal arises from stimulated anti-Stokes Raman scattering [55], [56] as illustrated in Figure S9 in the Supplementary Material. The stimulated Raman spectrum generated by the fundamental beam and QDs with size 1.94 nm is provided in Figure S8.

In the case of a 45 nm coated thickness, an intriguing observation is made regarding the nonlinear spectral broadening of the third harmonic, accompanied by an enhanced signal. The resonant harmonic (marked by the red line) shows significant amplification, particularly at higher intensities. However, for a coated thickness of 60 nm, as depicted in Figure 3(d), the resonant component is once again absent. Consequently, the harmonic at *hν* = 3.61 eV becomes most prominent at a specific thickness of the QD films.

The observed changes in spectral broadening and intensities apply to all CdTe quantum dots with sizes ranging from 1.94 to 2.16 nm, as listed in Table 1. The generated harmonic spectra for CdTe quantum dots synthesized with reaction times of 1 h (*d* = 2.01 nm), 2 h (*d* = 2.08 nm), and 4 h (*d* = 2.16 nm) are presented in the Supporting Information Figures S12, S13, and S14(a)–(d), respectively. In all three Figures S12–S14(a) and (b), the spectra exhibit a similar pattern to that shown in Figure 3(a) and (b). However, in (c), the situation differs slightly, although it still resembles the case in Figure 3(c). The resonant component emerges at a specific film thickness of 45 nm, while it remains absent in the other three thicknesses. Thus, the appearance of the resonant harmonic in Figures 3(c) and S12–S14(c), and its absence in the (a), (b), and (d) spectra, suggests that the appropriate CdTe QD coating thickness enables controlled spectral shaping through the manipulation of QD layer thickness and band dispersion. This highlights the potential to achieve specific spectral features through precise tuning of QD properties.

In Figure S11(c) of the Supplementary Material, the maximum yield of the third harmonic spectra from borosilicate glass plasma is compared to the maximum yield obtained using coated quantum dots with sizes ranging from 2–4 nm. Notably, for the largest and smallest quantum dots, the peak intensities are 17.5 and 10 times greater, respectively, than those from the bare substrate. However, a closer examination reveals that the spectra are not identical. When smaller quantum dots are used, spectral broadening is observed, a feature absent when larger quantum dots are employed.

**Figure 4:**
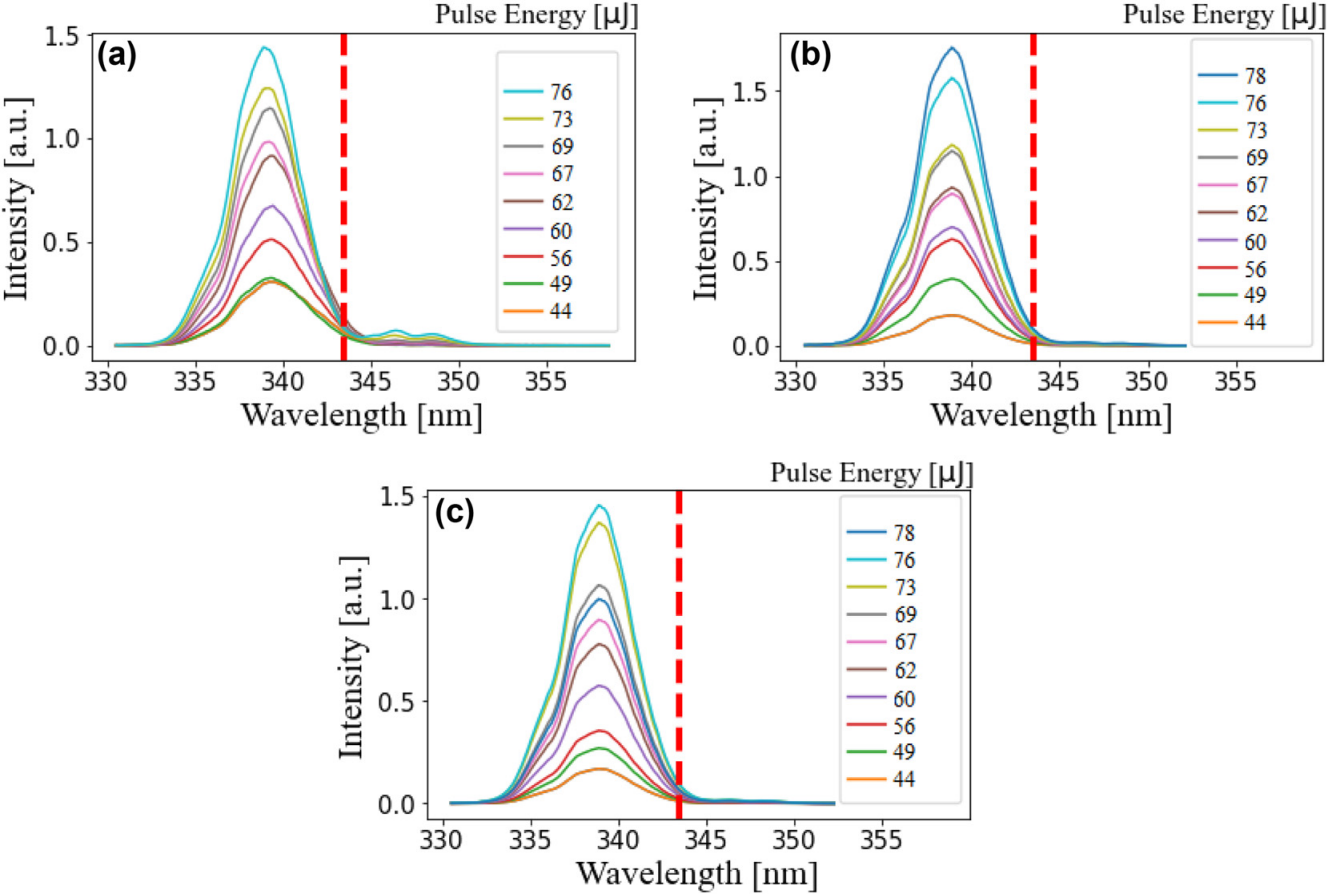
Generated third harmonic yield for CdTe with a size of 4.04 nm and for film thickness of (a) 10 nm, (b) 27 nm, (c) 45 nm and according to Figure S7(a)–(c). Here, the intensity of the third harmonic dropped after applying the third coating. The coupling to the TO modes disappeared almost completely. The spectrum exhibits a Gaussian like profile.

For the largest CdTe QDs, with a size of 4.04 nm and a bandgap of 1.77 eV (obtained after a reaction time of 48 h), the harmonic spectra are depicted in Figure 4(a)–(c) for three film thicknesses. Unlike the smaller dots, spectral broadening is entirely absent, regardless of the film thickness. The bandwidth of the spectrum is comparable to that of the original borosilicate glass and the spectra shown in Figure S16. Notably, the individual modes observed in the other spectra are no longer present. Instead, the spectrum adopts a Gaussian-like profile with a maximum peak intensity of 1.75 at a center wavelength of 339 nm, although the previous peaks can still be identified as small shoulders. This lack of spectral broadening suggests a size-dependent or quantum confinement effect achieved at certain film thicknesses. Furthermore, the modulation of the resonant component is absent across all thicknesses for QDs with a diameter of 4.04 nm.

For intermediate sizes of the CdTe QDs, a different behavior is observed. Figure S15(a)–(d) show the harmonic spectra for CdTe QDs with a reaction time of 6 h, characterized by a core size of 2.3 nm and a band gap of *E*_*g*_ = 2.25 eV. Significant changes compared to the previous spectra depicted in Figures 3, 4, and S12–S14 in the Supplementary Material are observed. A notable observation is the broadening of the spectrum compared to the original spectrum in Figure S11(a). Further additional modes are present, and a slightly larger peak intensity compared to the smaller dots is achieved in Figure S15(c), accompanied by a spectral bandwidth, which is the same as for the first QD used in Figure 3(c). Indeed, the spectral broadening observed in Figure S15 does not seem to be solely dependent on the coated thickness but rather appears to be influenced by the size of the CdTe quantum dots themselves. The different thicknesses in this case mainly affect the intensity of the harmonics. However, the resonant harmonic at 343 nm appears modulated for all thicknesses but does not show a peak at a film thickness of 45 nm as in the previously discussed spectra.

In the case of CdTe QDs synthesized with a reaction time of 24 h, a band gap of 1.97 eV, and a size of 2.95 nm, depicted in Figure S16, the spectral broadening observed in Figure S15 is significantly reduced. The spectra in Figure S16(a)–(d) exhibit narrower bandwidths, comparable to the original borosilicate glass spectrum, with a full width at half maximum (FWHM) of approximately 7 nm. The individual modes that appeared in the previous spectra are still present, although slightly suppressed in terms of spectral bandwidth. Nevertheless, peak intensities of 1.45 are achieved in this case, highlighting a trend of increasing peak intensity with larger quantum dot sizes. While the coupling to the LO modes of CdTe has completely disappeared, the coupling to the *E*_1_ and *A*_1_ modes persists. Theoretical studies [49] have shown that the electron–phonon coupling constant *g* decreases with increasing quantum dot size, and experimental observations reveal similar size-dependent behavior when a critical size is reached [50], [51]. Notably, the suppression of the resonant harmonic is absent across all film thicknesses for the QD size of *d* = 2.95 nm (Figure S16).

### 4.2 Discussion

In Section 2, we derived an expression for the third order susceptibility and the resonant part of the third harmonic. The gain of the resonant third harmonic is modulated according to eq. (9) by the phase mismatch Δ*k* = *k*_3_ − 3*k*_1_ of the resonant third harmonic and the fundamental beam. The phase mismatch can be obtained through the refractive index of bulk CdTe where *n*_3*ω*_ = 3.1 and *n*_1*ω*_ = 2.7 [57]. Thus, the bulk phase mismatch is derived as $\Delta k_{\text{b}}=\frac{3\omega }{c}\left(n_{3\omega }-n_{1\omega }\right)\approx 1.2\cdot 10^{4}\;cm^{-1}$ . This would result in a coherence length of $L_{c}=\frac{\pi }{2\Delta k}\approx 1.3\;\mu m$ for 3*ω*. However, the coherence length obtained from the thickness measurement reveal a coherence length of *L*_*c*_ ≈ 45 nm, which would require a phase mismatch of the electron–hole plasma of Δ*k*_p_ ≈ 3.5 × 10^5^ cm^−1^. Thus, there has to be a change of the refractive index due the density of free electrons *n*_*e*_ in the conduction band during the optical pulse duration. The detailed description is provided in the Supplementary Material. The refractive index of electron–hole plasma is given by

(10) $$n_{p}=\sqrt{1-\frac{\omega_{p}^{2}}{\omega_{i}^{2}}}\approx 1-\frac{\omega_{p}^{2}}{2\omega_{i}^{2}},$$

where $\omega_{p}=\frac{n_{e}e^{2}}{\epsilon_{0}m^{2}}$ is the plasma frequency and *ω*_*i*_ is the frequency of the *i*th harmonic. Assuming the above mentioned phase mismatch of Δ*k* ≈ 3.5 × 10^5^ cm^−1^, an electron density of *n*_*e*_ ≈ 10^21^ cm^−3^ can be estimated. This matches literature values for peak intensities of $I\propto 10^{13}\;\frac{W}{cm^{2}}$ [58]. The generated plasma frequency would be *ω*_*p*_ ≈ 5–6 THz and thus $\omega_{p}^{2}\ll \omega_{i}^{2}$ and the approximation in eq. (10) is valid. Since Δ*k*_b_ ≪ Δ*k*_p_, eq. (7) can be expanded around Δ*k*_b_, which is valid at *z* = 0

(11) $$\begin{array}{ll} \sin\left(\Delta kz\right) & \approx \sin\left(\Delta k_{\text{b}}z\right)+z\left(\Delta k_{\text{p}}-\Delta k_{\text{b}}\right)\cos\left(\Delta k_{\text{b}}z\right)\\ & \approx z\Delta k_{\text{p}}\cos\left(\Delta k_{\text{b}}z\right). \end{array}$$

Note, that the approximation in eq. (11) is only valid for small *z* (*L*_*c*,*p*_ ≪ *L*_*c*,*b*_). The ratio of Δ*k*_b_ and Δ*k*_p_ will be at least one order of magnitude and thus we can rewrite eq. (11)

(12) $$\sin\left(\Delta kz\right)\approx z\;\Delta k_{\text{p}}\cos\left(\frac{\Delta k_{\text{p}}}{q}\;z\right),$$

where *q* = Δ*k*_p_/Δ*k*_b_. It is worth noting that the ratio between the bulk and plasma phase mismatch is negative. Since the cosine is a symmetrical function, this will have no influence on the equation. Using eq. (12), we can rewrite eq. (9) to

(13) $$\begin{array}{ll} E\left(\omega_{3},z,t\right) & \propto \chi_{\text{eff}}^{\left(3\right)}\left(-3\omega ,\omega ,\omega ,\omega \right)\;E^{3}\left(\omega ,z,t\right)\\ & \;\times z\;\Delta k_{\text{p}}\cos\left(\frac{\Delta k_{\text{p}}}{q}\;z\right). \end{array}$$

The resonant nonlinear susceptibility $\chi_{\text{eff}}^{\left(3\right)}$ is determined by fitting eq. (9) to the power-dependent gain of the resonant harmonic at 343 nm for all film thicknesses. The corresponding fits are shown in Figure S19 in the Supplementary Material. The normalized $\chi_{\text{eff}}^{\left(3\right)}$ values are further analyzed by fitting them as a function of film thickness using eq. (13). For QDs of sizes between 1.94 and 2.16 nm a ratio of *q* = 20 and for the bigger dots with a size of *d* = 2.30 nm a ratio of *q* = 50 is found, which indicates a higher electron density in the conduction band.

**Figure 5:**
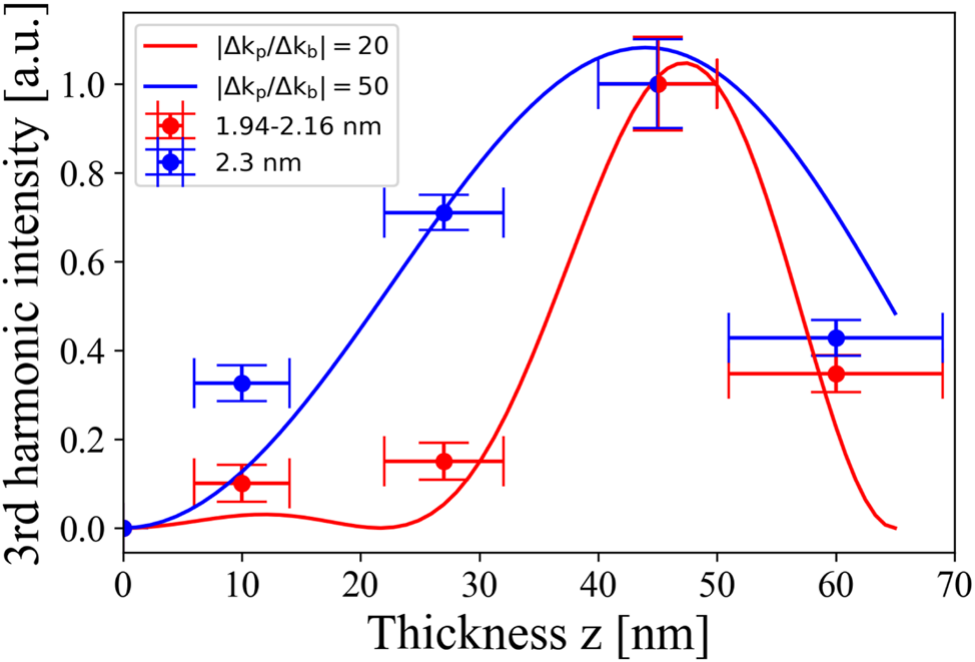
Coherent control of the resonant third harmonic intensity at *ℏω*_3_ = 343 nm is demonstrated. The normalized value of $\chi_{\text{eff}}^{\left(3\right)}$ , obtained from the power-dependent fit based on eq. (9) and Figure S19, is plotted as a function of film thickness.

Thus, the resonant third harmonic at *λ*_3_ = 343 nm can be modulated by coating different thicknesses of CdTe QDs. Especially the smaller dots are good candidates to control coherently the resonant part. In particular, the interference process occurs right at the beginning of the first monolayers of the coated QD films for the smaller dots (red graph, Figure 5). The interference is modulated by the phase mismatch between the resonant third harmonic and the fundamental when the film thickness is changed, as expected according eq. (9). The blue graph in Figure 5 shows that this process occurs also for a CdTe QD diameter of 2.30 nm, but the resonant harmonic appears not as a prominent peak, as can be seen in Figure S15 in the Supplementary Information and as described earlier. The modulation process itself thus occurs only for the smaller dots. This is a consequence of the different behavior of electrons in the conduction band with respect to the nonlinear susceptibility, which have to play a crucial role, since the phase mismatch is caused by the electrons in the conduction band. Hence, the value for *χ* ^(3)^ should also depend on the contribution of these electrons.

In Figure S18, we showed that the maximum value for *χ* ^(3)^ is strongly dependent on the size. The small dots (*d* < 2.30 nm) are characterized by the resonant and the bigger dots (*d* > 2.30 nm) by the nonresonant contribution of the free electrons to the susceptibility. This supports our corroboration that free electrons cause the phase mismatch and, therefore, the thickness-dependent interference between the fundamental and the third harmonic according to eq. (13). It also explains that the interference process only occurs for the smaller dots. The missing of the resonant third harmonic for the dots of sizes of 2.95 nm and 4.04 nm in Figures S16(a)–(d) and 4(a)–(c) is a consequence of the nonresonant contributions to *χ* ^(3)^ (see Sections 3 and 8.1 in the Supplementary Material). For a size of 2.30 nm, the resonant component is neither fully suppressed nor clearly visible as a distinct subpeak, as shown in Figure S15(a)–(d). This is due to the intermediate contributions of both the resonant and nonresonant components. In general, these subpeaks can be attributed to CdTe LO and TO phonon modes, which exhibit the expected size dependence in line with previous studies [49], [50], [51]. These phonon modes are essential for facilitating dipole coupling between the Γ point and the *X* point, leading to the generation of blue-shifted radiation.

## 5 Conclusions

In this work, we presented a new method for the coherent control of a photo-induced current in solid material by internally generated fields using different thicknesses of CdTe quantum dots with sizes around 2 nm. This could pave the way for new applications in ultrafast switching of nanophotonic devices, which uses quantum interference.

The direct band gap of bulk CdTe at the *L* point at 3.6 eV [44], [45], [46], [47] allows a resonant three-photon transition with the fundamental wavelength of 1,030 nm, which gives rise to quantum interference effects [23], [24], [27], [30], [31]. By varying the film thicknesses of CdTe quantum dots (QDs), the phase relationship between the external fundamental wavelength and the internally generated third harmonic can be controlled. This leads to either a suppression or significant enhancement of the resonant third harmonic, while the nonresonant components remain largely unaffected. This resonant behavior is specifically observed in QDs with diameters ranging from 1.94 nm to 2.16 nm. Theoretical calculations suggest that the third-order susceptibility tensor *χ* ^(3)^ is modulated due to phase mismatch between the external and internal fields when the seed pulse is tuned near the three-photon resonance. High peak intensities increase the number of conduction band electrons, leading to a temporary change in the refractive index of CdTe, which reduces the coherence length. This enables the use of nanometer-scale thicknesses to coherently control the resonant third harmonic intensity, making the process promising for nanophotonic applications.

Interestingly, this behavior is absent in the fifth harmonic, which supports our explanation, as the fifth harmonic is far from any resonant transition in CdTe. Larger quantum dots also do not exhibit this resonant behavior. Their harmonic yield is dominated by nonresonant intraband mechanisms of conduction band electrons contributing to the third-order nonlinear susceptibility, *χ* ^(3)^, as predicted by the theory of nanoparticles [39]. This may also explain the lack of quantum interference in larger dots.

In summary, we coherently control the appearance of the resonant third harmonic in a strong dipole coupled three-photon resonant transition by choosing the right size and thickness of CdTe QDs. The well-known exciton in CdTe at the *L* point has a strong Coulomb coupling providing excellent conditions for quantum interference. In particular, all materials that exhibit saddle type critical points including alkali halides [59], III–V, and II–VI semiconductors [46] are possible candidates for such a behavior and, therefore, opening new opportunities to the design of nonlinear opto-electronic devices.

## Supplementary Material

Supplementary Material Details

Supplementary Material Details
