# Supplementary material for: Quantum control in size selected semiconductor quantum dot thin films
Source: Nanophotonics. 2025 Jan 16;14(2):229–39. doi: 10.1515/nanoph-2024-0529 (PMC11806504; doi:10.1515/nanoph-2024-0529)
Supplement: Supplementary file 1 — Supplementary Material Details [file j_nanoph-2024-0529_suppl_001.pdf]

Research Article

Victor Kärcher*, Tobias Reiker, Pedro F.G.M. da Costa, Andrea S.S. de Camargo, and Helmut Zacharias

Quantum Control in Size Selected Semiconductor Quantum Dot Thin Films-Supplemental Material

1 Quantum interference

In this section we will provide a detailed calculation to describe our results. We will use the semiclassical approach of a particle interacting with an external field by solving the time dependent Schrödinger equation for different power dependencies n. In other words by just applying Fermi's golden rule, we calculated the transition probabilities for a one ($n = 1$) and a three ($n = 3$) photon process. This transition probabilities are then connected to the third order non-linear susceptibility tensor which is modulated by the phase relation of the non-zero phase mismatch Δk of the fundamental and the third harmonic. The propagation characteristics are then gained by solving Maxwell's equations with a non-linear source term. In the whole description we will omit the operator and vector notation.

Consider the time dependent problem with the Hamiltonian

$$H = H_0 + \lambda H_{int}(t), \tag{S1}$$

where H_0 is the unperturbed, $H_{int}(t)$ the perturbed operator, and λ is the coupling constant. The unperturbed solution is

$$H_0 \left|n\right\rangle = \hbar\omega_n \left|n\right\rangle, \tag{S2}$$

$\hbar$ is the reduced Planck's constant and ω_n are the eigenfrequencies of state $|n\rangle$.

The perturbation H_{int} starts at $t = 0$ and we have to solve the time dependent Schrödinger equation

$$i\hbar\frac{\partial}{\partial t}\left|\psi(t)\right\rangle = \left[H_0 + \lambda H_{int}(t)\right]\left|\psi(t)\right\rangle \tag{S3}$$

with

$$\left|\psi(t)\right\rangle = \sum_m c_m(t)\exp(-i\omega_m t)\left|m\right\rangle, \tag{S4}$$

***Corresponding author: Victor Kärcher,** Center for Soft Nanoscience, University of Münster, 48149 Münster, Germany, v_kaer01@uni-muenster.de; 0009-0002-1622-9857

Tobias Reiker, Center for Soft Nanoscience, University of Münster, 48149 Münster, Germany, t_reik01@uni-muenster.de; 0000-0001-8720-5882

Helmut Zacharias, Center for Soft Nanoscience, University of Münster, 48149 Münster, Germany, hzach@uni-muenster.de; 0000-0001-9807-1103

Pedro F.G.M. da Costa, São Carlos Institute of Physics, University of São Paulo, São Carlos - SP 13566-590, Brazil, costapedro@usp.br; 0000-0003-4600-7817

Andrea S.S. de Camargo, Federal Institute for Materials Research and Testing (BAM), 12489 Berlin, Germany,andrea.camargo@bam.de,0000-0001-8352-2573

Andrea S.S. de Camargo, Friedrich-Schiller University Jena (FSU), 07743 Jena, Germany

where $|c_m(t)|^2$ is the probability of finding a particle in state $|m\rangle$ at time t and $\psi(t)$ is the particles wave function. Solving the Schrödinger equation after multiplying with $\langle m|$ from the left side gives

$$i\hbar\frac{\partial}{\partial t}c_m(t) = \lambda\sum_m c_m(t)\exp(-i\omega_m t)\langle m|H_{int}|m\rangle . \tag{S5}$$

Now, we can expand $c_m(t)$ in terms of the power dependence of the coupling parameter λ^n

$$c_m(t) = \lambda^0 c_m^{(0)}(t) + \lambda^1 c_m^{(1)}(t) + \lambda^2 c_m^{(2)}(t) + \lambda^3 c_m^{(3)}(t) + ... \tag{S6}$$

The initial conditions are

$$c_m^{(0)}(t) = \delta_{nm} \text{ and } c_m^{(1)} = c_m^{(2)} = ... = 0, \tag{S7}$$

where δ_{nm} is the Kronecker delta. Thus, the zero order solution to the Schrödinger equation gives

$$\frac{\partial}{\partial t}c_m^{(0)}(t) = 0 \Rightarrow c_m^{(0)}(t) = \delta_{m,n}. \tag{S8}$$

The first order gives

$$\frac{\partial}{\partial t}c_m^{(1)}(t) = \sum_{m'} c_{m'}^{(1)}(t)\exp(i\omega_{m,m'}t)\langle m|H_{int}|m'\rangle \overset{c_m'^{(0)}(t)=\delta_{m',n}}{=} \exp(i\omega_{m,m'}t)\langle m|H_{int}|n\rangle . \tag{S9}$$

Thus, we have

$$c_m^{(1)}(t) = \frac{i}{\hbar}\int_0^t dt' exp(i\omega_{m,m'}t')\langle m|H_{int}|n\rangle \tag{S10}$$

Writing this equation for n-th order gives the time operator

$$U(t,t_0) = \sum_n\left(\frac{i}{\hbar}\right)^n\int_0^t dt_1 H_{int}(t_1)\int_0^{t_1} dt_2 H_{int}(t_2)\cdots\int_0^{t_{n-1}} dt_n H_{int}(t_n) \tag{S11}$$

The Hamiltonian for a particle in an external field in semi classical picture is given by

$$H = \frac{1}{2m}\left(p - \frac{e}{c}A(t)\right)^2 + U(r), \tag{S12}$$

with mass m, momentum p, charge e, light velocity c, external field A and potential U. The interaction Hamiltonian is

$$H_{int}(t) = -\frac{e}{mc}pA(t). \tag{S13}$$

The external field can be written as

$$A(z,t) = A_\omega\exp\left(-(\omega+i\Gamma)t+kz\right) + A_\omega^*\exp\left((\omega-i\Gamma)t-kz\right)+$$

$$+A_{\omega_3}\exp\left(-(\omega_3+3i\Gamma)t+k_3z\right) + A_{\omega_3}^*\exp\left((\omega_3-3i\Gamma)t-k_3z\right), \tag{S14}$$

with $\omega = \omega_1$ and $k = k_1$ the fundamental frequency and wave number, ω_3 and k_3 denote the third harmonic frequency and wave number, respectively, and Γ denotes the linewidth. Now we can calculated the transition amplitude $c_m^{(n)}(t)$ for one photon from the initial state i to the final state f at position z for frequency ω_3

$$c_f^{(1)}(z,t) = -\frac{e}{\hbar cm}p_{fi}A_{\omega_3}\frac{e^{-i(\omega_3-\omega_{fi}+i\Gamma)t}}{\omega_3-\omega_{fi}+i\Gamma}e^{ik_3z}, \text{with } p_{fi} = \langle f|p|i\rangle \tag{S15}$$

and the three photon transition amplitude for ω

$$c_f^{(3)}(z,t) = -\left(\frac{e}{\hbar cm}\right)^3\sum_{l,j}\frac{p_{fl}A_\omega p_{lj}A_\omega p_{ji}A_\omega}{(\omega-\omega_{ji})(2\omega-\omega_{li})}\frac{e^{-i(3\omega-\omega_{fi}+3i\Gamma)t}}{(3\omega-\omega_{fi}+3i\Gamma)}e^{3ikz}. \tag{S16}$$

Due to symmetry consideration this term is only nonzero for non-centro symmetric crystal structures when summing over the whole Brillouin zone since $p_{fi}(k_{el}) = -p_{fi}(-k_{el})$. Our quantum dots have zinc blende crystal structure and therefore this term will be non zero in our case.

In order to calculate the transition rate T we have to take the time derivative of the square of the sum of the one and three photon transition probabilities

$$T = \frac{d}{dt}|c_f^{(1)}(z,t) + c_f^{(3)}(z,t)|^2 \tag{S17}$$

and thus

$$T = \frac{d}{dt}\left(|c_f^{(1)}(z,t)|^2 + |c_f^{(3)}(z,t)|^2 + c_f^{(1)}(z,t)(c_f^{(3)}(z,t))^* + c_f^{(3)}(z,t)(c_f^{(1)}(z,t))^*\right) \tag{S18}$$

It is directly obvious that the term consists of two interference terms. The first one is

$$c_f^{(1)}(z,t)(c_f^{(3)}(z,t))^* = \left(\frac{e}{\hbar mc}\right)^4 \sum_{f,l,j,i} \frac{p_{if}A_{\omega_3}p_{fl}A_{\omega}p_{lj}A_{\omega}p_{ji}A_{\omega}}{(\omega-\omega_{ji})(2\omega-\omega_{li})}\times$$

$$\times\frac{e^{i(3\omega-\omega_{fi}-3i\Gamma)t}}{(3\omega-\omega_{fi}-3i\Gamma)}e^{-3ikz}\frac{e^{-i(\omega_3-\omega_{fi}+i\Gamma)t}}{\omega_3-\omega_{fi}+i\Gamma}e^{ik_3z} \tag{S19}$$

We can rewrite this term using $p = im\omega x$ and obtain

$$c_f^{(1)}(z,t)(c_f^{(3)}(z,t))^* = \left(\frac{e}{\hbar mc}\right)^4 (im\omega)^4 \sum_{f,l,j,i} \frac{x_{if}A_{\omega_3}x_{fl}A_{\omega}x_{lj}A_{\omega}x_{ji}A_{\omega}}{(\omega-\omega_{ji})(2\omega-\omega_{li})}\times$$

$$\times\frac{e^{i(3\omega-\omega_{fi}-3i\Gamma)t}}{(3\omega-\omega_{fi}-3i\Gamma)}e^{-3ikz}\frac{e^{-i(\omega_3-\omega_{fi}+i\Gamma)t}}{\omega_3-\omega_{fi}+i\Gamma}e^{ik_3z} \tag{S20}$$

Using the definition of the third order nonlinear susceptibility tensor $\chi^{(3)}$

$$\chi^{(3)}(-3\omega,\omega,\omega,\omega) = \frac{N}{\epsilon_0}\frac{e^4}{3!\hbar^3}\sum_{f,l,j,i}\frac{x_{if}x_{fl}x_{lj}x_{ji}}{(\omega-\omega_{ji})(2\omega-\omega_{li})(3\omega-\omega_{fi}-3i\Gamma)}, \tag{S21}$$

where N is the number of oscillators, ϵ_0 is the permeability, we can rewrite the interference term as

$$c_f^{(1)}(z,t)(c_f^{(3)}(z,t))^* = \left(\frac{N}{\epsilon_0}\frac{e^4}{3!\hbar^3}\right)^{-1}\left(\frac{e}{\hbar mc}\right)^4 (im\omega)^4 \chi^{(3)}(-3\omega,\omega,\omega,\omega)\times$$

$$\times A_{3\omega}A_{\omega}^3\frac{e^{i(\Delta\omega t-2i\Gamma t+\Delta kz)}}{\omega_3-\omega_{fi}+i\Gamma} \tag{S22}$$

with $\Delta\omega = \omega - \omega_3$ and $\Delta k = k_3 - 3k$. Now we can calculate the first time derivative at $t = 0$

$$\frac{d}{dt}c_f^{(1)}(z,t)(c_f^{(3)}(z,t))^*|_{t=0} = \left(\frac{N}{\epsilon_0}\frac{e^4}{3!\hbar^3}\right)^{-1}\left(\frac{e}{\hbar mc}\right)^4 (m\omega)^4 \chi^{(3)}(-3\omega,\omega,\omega,\omega)\times$$

$$\times\ A_{3\omega}A_{\omega}^3\frac{i(\Delta\omega-2i\Gamma)}{\omega_3-\omega_{fi}+i\Gamma}e^{i\Delta kz} \tag{S23}$$

When Γ approaches zero, the function becomes

$$\frac{d}{dt}c_f^{(1)}(z,t)(c_f^{(3)}(z,t))^*|_{t=0} = \left(\frac{N}{\epsilon_0}\frac{e^4}{3!\hbar^3}\right)^{-1}\left(\frac{e}{\hbar mc}\right)^4 (m\omega)^4 \chi^{(3)}(-3\omega,\omega,\omega,\omega)\times$$

$$\times A_{3\omega}A_{\omega}^3 ie^{i\Delta kz}\delta(\omega_{fi}-\omega_3) \tag{S24}$$

This is because $(3\omega - \omega_3 + 2i\Gamma)/(\omega_3 - \omega_{fi} + i\Gamma)$ can be written as real

$$\Re = \frac{(3\omega - \omega_3)(\omega_3 - \omega_{fi}) + 2\Gamma^2}{(\omega_3 - \omega_{fi})^2 + \Gamma^2} \tag{S25}$$

and imaginary part

$$\Im = \frac{2\Gamma(\omega_3 - \omega_{fi}) - (3\omega - \omega_3)\Gamma}{(\omega_3 - \omega_{fi})^2 + \Gamma^2}. \tag{S26}$$

In the limit $\Gamma \to 0$ only the real part remains with

$$\Re = \frac{3\omega - \omega_3}{\omega_3 - \omega_{fi}} \tag{S27}$$

For third harmonic generation $3\omega = \omega_3$ and thus, the numerator in eq. (S27) becomes zero. When the third harmonic is resonant with the transition $f \to i$, then $\omega_3 = \omega_{fi}$ and thus also the denominator becomes zero. After applying the rules of L'Hopital, the real part $\Re$ converges to -1. When the third harmonic is non-resonant to the transition $f \to i$, only the numerator becomes zero and thus, the complete interference term vanishes. So $(3\omega - \omega_3 + 2i\Gamma)/(\omega_3 - \omega_{fi} + i\Gamma) \to \delta(\omega_{fi} - \omega_3)$ as $\Gamma \to 0$.

With

$$e^{i\Delta kz} = \cos(\Delta kz) + i\sin(\Delta kz) \tag{S28}$$

the real part of equation (S24) becomes proportional to $\sin(\Delta kz)$, since there is an i left from the time derivative. Thus we can define a $\chi^{(3)}_{eff}(-3\omega, \omega, \omega, \omega)$ with

$$\Re(\chi^{(3)}_{eff}(-3\omega, \omega, \omega, \omega)) = \chi^{(3)}(-3\omega, \omega, \omega, \omega)\sin(\Delta kz) \tag{S29}$$

and

$$\Im(\chi^{(3)}_{eff}(-3\omega, \omega, \omega, \omega)) = \chi^{(3)}(-3\omega, \omega, \omega, \omega)\cos(\Delta kz) \tag{S30}$$

so for $z = 0$ the $\Re(\chi^{(3)}_{eff}(-3\omega, \omega, \omega, \omega))$ becomes zero and quantum interference is expected. The 3rd harmonic will show different absorbing and amplification behavior along the z direction in the sample and will also depend on the phase relation between the first and third harmonic.

Now, we can write down the Maxwell equation

$$\nabla \times \nabla \times E(\omega_3, z, t) - \epsilon_3 \frac{\omega_3^2}{c^2} E(\omega_3, z, t) = 4\pi \frac{\omega_3^2}{c^2} P^{\text{NLS}}(-3\omega, \omega, \omega, \omega, z, t) \tag{S31}$$

with $E = (-1/c)(d/dt)A$ according to eq. (S14) and the external non-linear driving source term $P^{\text{NLS}}(-3\omega, \omega, \omega, \omega, z, t)$. $\nabla \times \nabla \times E(\omega_3, z, t) = k_3^2 E(\omega_3, z, t)$ with $k_3 = \omega_3/c$. The solution to this equation is

$$E(\omega_3, z, t) = E_\omega e^{i(3kz - 3\omega t)} + E_{\omega_3} e^{i(k_3 z - \omega_3 t)} + cc. \tag{S32}$$

The first term E_ω propagates with c/n_1 and the second term E_{ω_3} propagates with c/n_3 with $n_{1,3} = \sqrt{\epsilon_{1,3}}$. In the strongly absorbing case at $z = 0$ the second term becomes zero according to eq. (S30), which is the free wave solution of the homogeneous wave equation. So only the stimulated emission with constant phase relation to the fundamental remains. Thus, the solution becomes

$$E(\omega_3, z, t) = \frac{4\pi}{\epsilon_1 - \epsilon_3} P^{\text{NLS}}(-3\omega, \omega, \omega, \omega, z, t) \tag{S33}$$

and

$$\begin{aligned} P^{\text{NLS}}(-3\omega, \omega, \omega, \omega, z, t) &= \Re(\chi^{(3)}_{eff}(-3\omega, \omega, \omega, \omega))E^3(\omega, z, t) \\ &= \chi^{(3)}(-3\omega, \omega, \omega, \omega)\sin(\Delta kz)E^3(\omega, z, t) \end{aligned} \tag{S34}$$

Thus, the gain of the third harmonic will be $\propto \sin(\Delta k z)$ for $\Delta k \neq 0$ and for a constant phase relation between the fundamental and the third harmonic. For a given distance $z = z_T$, the imaginary part of $\chi^{(3)}_{eff}(-3\omega, \omega, \omega, \omega)$ becomes zero and the free wave propagation becomes possible and thus the third harmonic will be generated. Therefore the yield of the third harmonic which is resonant with the transition $f \rightarrow i$ will be dependent on the samples thickness z.

2 CdTe quantum dot-size measurements

The characteristics of nano sized particles is the confinement of energy bands, an effect known as quantum confinement. Quantum confinement occurs when the size of the dot is well below the Bohr radius. The measured electronic Bohr radius of CdTe is 7 nm [5] and thus quantum confinement is valid. The effect is characterized by an increase in the band gap with decreasing size. The energy difference to its bulk counterpart at the Γ point is given by [6]

$$\Delta E_g = \frac{\hbar\,\pi^2}{2\mu\, d^2} - \frac{1.8\, e^2}{\epsilon\, d}, \tag{S35}$$

where μ is the reduced mass of the hole and the electron, $\epsilon = \epsilon_0\,\epsilon_b$ is the permittivity of the bulk material. The first term is connected to the localization of the particle due to quantum confinement and the second term is a consequence of Coulomb interaction. Fig. S2 shows the size dependent energy shift according to eq. (S35). High resolution transmission electron microscope (HRTEM) images for the QDs generated with a reaction time of 24h are depicted in Fig. S4 at two different magnifications. These pictures indicate that these QDs have sizes of about 3 nm which supports the QD size obtained with the ABS method provided in Tab. S2 Fig. S1 shows the fluorescence quantum yield for the QDs ranging from the sizes 1.94-2.95 nm. Linear quantum yields of 0.25% for the smallest and 17.5% for the QD diameter of 2.95 nm are obtained.

The TO modes are schematically shown in Fig. S3 a) and the size dependent absorption spectra of the QDs are shown in Fig. S3 b).

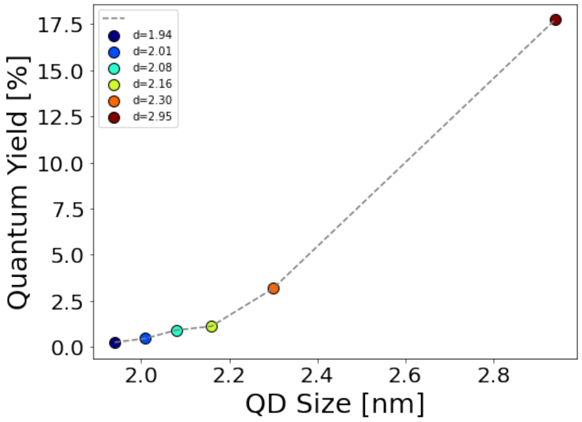

Fig. S1: Fluorescence quantum yield for QDs ranging from the sizes 1.94-2.95 nm. The Excitation wavelength is 405 nm

Tab. S1: Phonon modes of bulk CdTe [7].

Phonon mode	[cm^{-1}]
A_1 (TO)	123
E_1 (TO)	142
LO	166
2LO	333

Tab. S2: Characteristics of the CdTe quantum dots used in this work. The first column displays the reaction time, the second provides the band gap E_g at the Γ point (luminescence emission maximum). The third column shows the diameter derived with the absorption method (ABS) and the fourth shows the diameter measured with dynamic light scattering (DLS).

QD	E_g [eV]	d[nm] (ABS)	d[nm] (DLS)
0.5 h	2.54	1.94	-
1 h	2.47	2.01	2.9
2 h	2.41	2.08	3.03
4 h	2.34	2.16	3.03
6 h	2.25	2.30	3.33
24 h	1.97	2.95	3.94
48 h	1.77	4.04	-

2.1 Chemicals

Below we show the chemicals and their purity used for the generation of the QDs
- cadmium chloride hemipentahydrate – $CdCl_2$ $2.5H_2O$ (Sigma-Aldrich 99,995%)
- 3-mercaptopropionic acid – $HSCH_2CH_2COOH$ (Sigma-Aldrich $\geq$ 99.0% HPLC)
- sodium borohydride – $NaBH_4$ (Sigma-Aldrich $\geq$ 98.0%)
- metallic tellurium – Te^0 (Sigma-Aldrich 99.997%)
- sodium hydroxide – NaOH (Êxodo 97%)
- ultrapure water (Milli-Q®)

3 Nonlinear susceptibility of nanoparticles

The optical responses of nano sized particles differ strongly from their bulk counterpart. The confinement of electrons leads to a quantization of energy bands and thus to the formation of plasmonic resonances. The nonlinear optical response will be not only dependent on the transition from the conduction band to the valence band but also on the discrete transitions within the conduction band. Hence, the optical response will be modulated by a local field enhancement factor $f(\omega, d)$. The local field E_0 will be enhanced at the nanoparticle as [8, 9]

$$E_i = f(\omega, d)\, E_0, \text{ where } f(\omega, d) = \frac{3\epsilon_h}{\epsilon_n + 2\epsilon_h}, \tag{S36}$$

where ϵ_h describes the permittivity of the host medium and ϵ_n is that of the nano particle. The permittivity of the nanoparticle can be written as [8, 10]

$$\epsilon_n = \underbrace{\epsilon_n^{(1)}}_{\propto d} + 12\pi \underbrace{\chi_n^{(3)}(-3\omega, \omega, \omega, \omega)}_{\propto d^2 + d^{-3}} \underbrace{|f(\omega, d|^2}_{\propto d^2} \underbrace{I_0}_{\propto d E_0^2} \tag{S37}$$

where $\chi_n^{(3)}(-3\omega, \omega, \omega, \omega)$ denotes the third order susceptibility tensor of the nanoparticle itself and $I_0 = 1/2\epsilon_n^{(1)}\, c\, E_0^2$ the intensity of the driving field. The linear permittivity $\epsilon_n^{(1)}$ is directly connected to

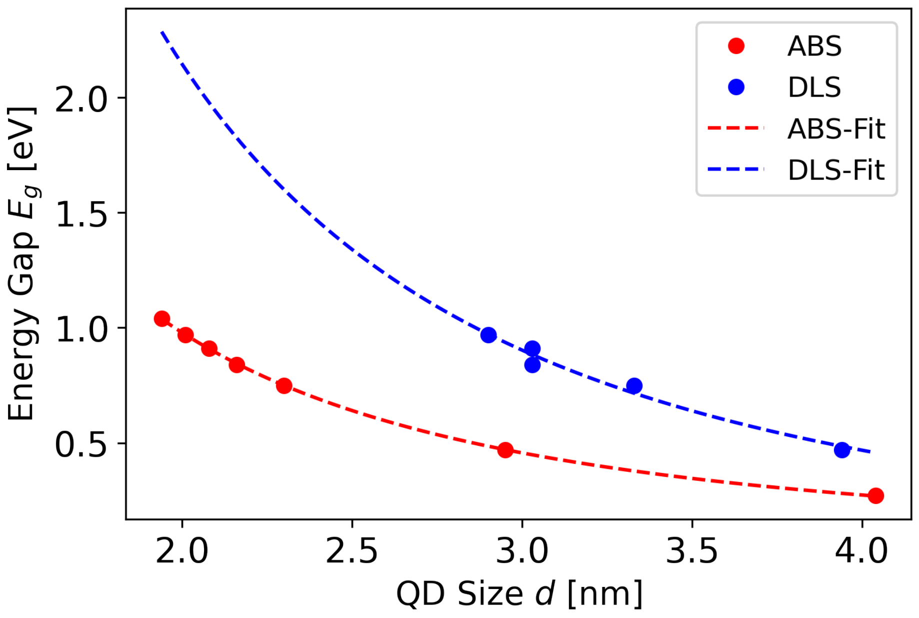

Fig. S2: Size dependent band gap difference of the QD band gap $E_{g,q}$ to the bulk band gap $E_{g,b} = 1.6\,\text{eV}$ at the Γ point according to eq. (S35) for the ABS measurement (red) and for the DLS measurement (blue).

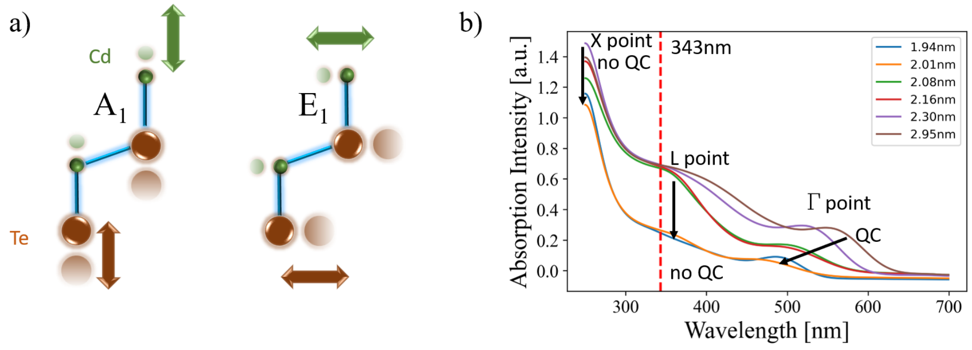

Fig. S3: a) Schematic representation of the A_1 and E_1 modes of the transverse optical phonons of CdTe. b) Absorption spectra for CdTe QDs of sizes 1.94 nm to 2.95 nm. Quantum confinement (QC) only occurs at the Γ point but not at the X and L point (no blue shift in absorption with decreasing size).

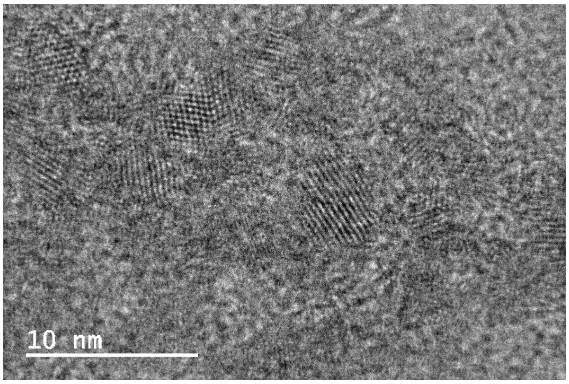

(a) HRTEM image showing the details with a scale of 10 nm.

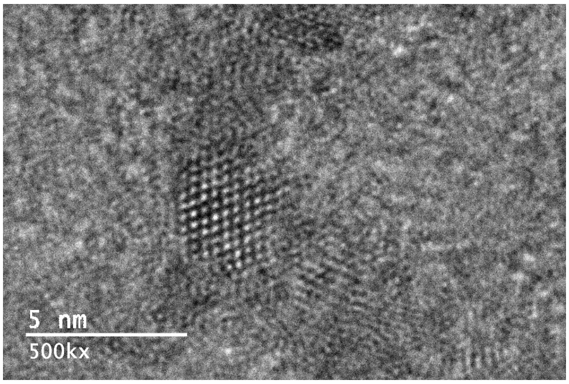

(b) HRTEM image showing the details with a scale of 5 nm. The QDs have sizes around 3 nm.

Fig. S4: HRTEM images of QDs with a size of $2.95\,\text{nm}$

the first order susceptibility which is gained by the square of the one-photon transition rate according to eq. (S20). The linear susceptibility is given by

$$\epsilon_n^{(1)} = 1 + \chi_n^{(1)} = 1 + \frac{N\,e^2}{\epsilon_0\,m}\frac{|p_{fi}|^2}{\omega_3 - \omega_{fi} + i\Gamma} \propto d. \tag{S38}$$

The square of the dipole matrix element is directly proportional to the QD diameter d ($|p_{fi}|^2 \propto d$) [11]. The third order susceptibility tensor scales to the fourth power of the dipole transition according to eq. (S21). The modulated nonlinear susceptibility is then given by [8, 9]

$$\chi_m^{(3)}(-3\omega,\omega,\omega,\omega) = f(\omega,d)^2\,|f(\omega,d)|^2\,\chi_n^{(3)}(-3\omega,\omega,\omega,\omega) \tag{S39}$$

However, due to eq. (S37) the field enhancement factor will decrease rapidly due to saturation of optical transitions at the high intensity regime and the field enhancement will become small and size independent for intensities above $I = 10^{13}\,\mathrm{W/cm^2}$ [8]. A qualitative dependence of the field enhancement factor according to eq. (S36) is depicted in Fig. S5. The third harmonic will solely depend on the susceptibility tensor of the nanoparticle itself which can be written as the sum over the non-resonant $\chi^{(3)}_{n,non-res}(-3\omega,\omega,\omega,\omega)$ and resonant contributions $\chi^{(3)}_{n,res}(-3\omega,\omega,\omega,\omega)$

$$\chi_n^{(3)}(-3\omega,\omega,\omega,\omega) = \chi^{(3)}_{n,non-res}(-3\omega,\omega,\omega,\omega) + \chi^{(3)}_{n,res}(-3\omega,\omega,\omega,\omega). \tag{S40}$$

The non-resonant part will dominate the larger dots, since $\chi^{(3)}_{n,non-res} \propto d^2$ and the resonant part will dominate for the smaller dots, since $\chi^{(3)}_{n,res} \propto d^{-3}$. Accordingly, the nonlinear susceptibility of the nanoparticle can be written as [8]

$$\chi_n^{(3)}(-3\omega,\omega,\omega,\omega) = \beta\,d^2 + \gamma\,d^{-3}, \tag{S41}$$

where β and γ are related to transition rates in the conduction band. Further the L point of CdTe is not only characterized by strong Coulomb coupling but also provides strong one- and three-photon absorption, which is required for quantum interference [12, 13]. Hence the nonlinear response will be modulated by the absorption coefficient [14, 15]

$$\alpha(\omega) = \alpha^{(1)}(\omega) + \alpha^{(3)}(\omega), \tag{S42}$$

in first and in third order respectively. The one-photon absorption is given by:

$$\alpha^{(1)}(\omega) = \Im\left(\omega\sqrt{\frac{\mu_0}{\epsilon_n^{(1)}}}\frac{N\,e^2}{\epsilon_0\,m}\frac{|p_{fi}|^2}{\omega_3 - \omega_{fi} + i\Gamma}\right) \propto \frac{|p_{fi}|^2}{\sqrt{\epsilon_n^{(1)}}} \propto \sqrt{d} \tag{S43}$$

and the nonlinear three-photon absorption is given by:

$$\alpha^{(3)}(\omega) = \Im\left(\omega\sqrt{\frac{\mu_0}{\epsilon_n^{(1)}}}E_0^2\frac{N\,e^4}{\epsilon_0\,m3!\hbar^3}\frac{|p_{fi}|^4}{\omega_3 - \omega_{fi} + i\Gamma}\right) \propto \frac{|p_{fi}|^4}{\sqrt{\epsilon_n^{(1)}}} \propto d^{3/2} \tag{S44}$$

Thus, the size depended nonlinear susceptibility will be modulated by the absorption coefficient according

$$\frac{\chi^{(3)}}{\alpha(\omega)} = \frac{\beta\,d^2 + \gamma\,d^{-3}}{\delta\sqrt{d} + \rho\,d^{3/2}} = \sqrt{I^{(3)}_{\max}}, \tag{S45}$$

in which δ and ρ provide the information of the one- and three-photon absorption and $I^{(3)}_{\max}$ is the maximum intensity of the resonant or non-resonant third harmonic.

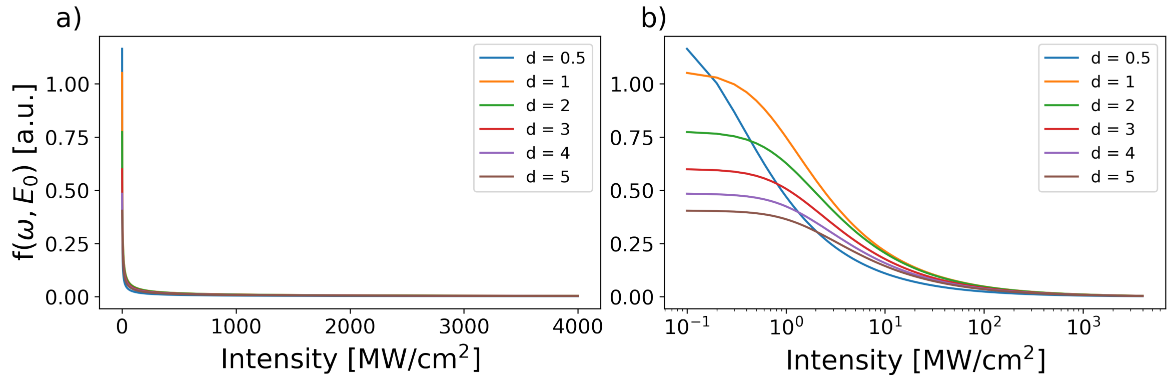

Fig. S5: Qualitative dependence of the field enhancement factor $f(\omega, E_0)$ on the intensity of the driving field on a linear scale in a) and on a logarithmic scale in b) for better visualization. The field enhancement factor becomes small and size independent for high intensities.

4 Borosilicate glass

The QDs were coated onto a glass wafer. Therefore, the characteristics of the target should also be discussed. Since we generate the harmonics in an insulator/semi conductor material, electrons and the light will interact with phonon modes leading to Raman Stokes and Anti-Stokes scattering which results in red and blue shifts of the spectrum or the appearance of new modes. The interaction may occur with longitudinal optical (LO) and transversal optical (TO) phonon modes. The latter is exemplary shown in Fig. S3 a). The Raman lines of borosilicate glass [2, 16] are shown in Tab. S3 with the corresponding shift for excitation at 343 nm and 206 nm.

Tab. S3: Table describing Raman Lines of borosilicate glass and the shift with respect to the third harmonic ($\lambda_3 = 343$ nm ,$\nu_3 = 29154\,\mathrm{cm}^{-1}$) and the fifth harmonic ($\lambda_5 = 206$ nm,$\nu_5 = 48544\,\mathrm{cm}^{-1}$) [2, 16]

Raman [cm^{-1}]	$\tilde{\nu}_3$ [cm^{-1}]	$\tilde{\lambda}_3$ [nm]	observed	$\tilde{\nu}_5$ [cm^{-1}]	$\tilde{\lambda}_5$ [nm]	observed
+607	29761	336	yes	49151	203.5	yes
-607	28547	350.3	no	47937	208.6	yes
+450	29604	338	yes	48994	204	no
-450	28704	348.4	no	48094	207.9	yes
+354	29466	338.9	yes	48898	204.5	no
-354	28800	347.2	no	48190	207.5	yes
+206	29360	340.6	yes	48750	205.1	no
-206	28948	345.5	yes	48338	206.9	yes
+130	29284	341.5	yes	48674	205.5	yes
-130	29024	344.5	yes	48414	206.5	no

5 Preparation and topography of the samples

The qualitative evaluation of the harmonic spectra relies on understanding the topography of the samples. To obtain the necessary information about the coated structures, an atomic force microscope (AFM) (BRUKER Nanoscope V using a Tespa V2 tip) was used. Fig. S6 shows the surface height variations of the uncoated target wafer, with a x and y length of 20 μm. The surface roughness is within a range of approximately ± 2 nm.

The samples were prepared by placing a 1 μl drop of CdTe quantum dots dissolved in water onto the glass wafer. The evaporation of the water took approximately 5 minutes. In a separate procedure, the sample was placed onto a commercial spin coater and spun at a rotational speed of 500 rpm for 5 minutes. This process was repeated four times for the third harmonic and two times for the fifth harmonic to achieve the desired coating thickness. The particle distribution for particles of about 2.95 nm average diameter is depicted in Fig. S7 a). The entire procedure was then repeated, doubling the number of repetitions for each subsequent coated thickness. Figs. S7 (a)-(d) provide examples of the subsequent coating thicknesses for the third harmonic with CdTe QDs of a diameter of 2.95 nm. It is evident from these pictures that the spin-coated QDs exhibit a grain like distribution rather than a uniform arrangement.

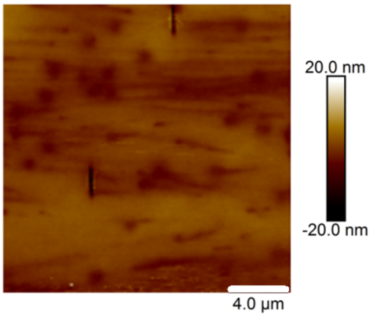

Fig. S6: AFM topography of the uncoated sample with a rms value of $\Delta h = 1.94$ nm

6 Stimulated Raman Spectrum

The figure clearly demonstrates that the anti-Stokes shifts dominate the process compared to the Stokes shifts.

As parameter of the different spectra traces the pulse energy of the fundamental ranging from 30 - 80 μJ, or intensities from (1.6 - 4.5) $\cdot 10^{13}$ W/cm^2. The modes of the host medium (borosilicate glass) are distinctly visible at approximately 450 cm^{-1} and 607 cm^{-1}. Additionally, the CdTe phonon modes, which lie between 120 cm^{-1} and 170 cm^{-1}, are evident adjacent to the 1020 nm peak on the high energy and low energy side, Anti-Stokes (AS) and Stokes (S) respectively. Fig. S9 displays schematically the process responsible for the generation of the off resonant harmonics ranging from 335 nm to 343 nm

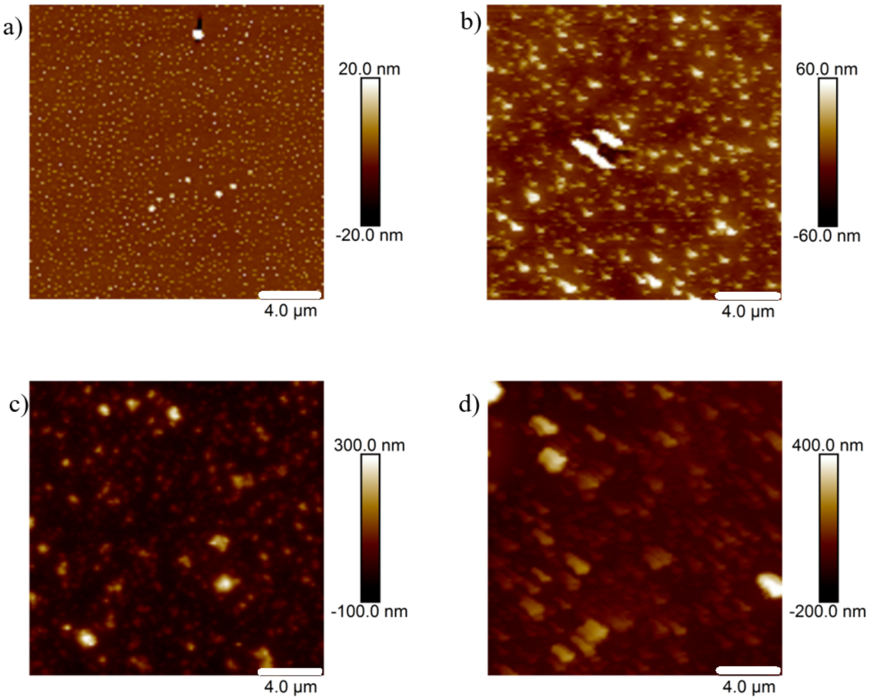

Fig. S7: AFM topography of the coated samples with CdTe quantum dots for different particle densities of about 2.95 nm diameter. In a) the thinnest coating is shown. The average grain size in transverse direction is $d_g = 183 \pm 33$ nm and the average grain height is $h_g = 8 \pm 4$ nm. b) shows the second coating with $d_g = 414 \pm 263$ nm and $h_g = 27 \pm 17$ nm, c) shows the third coating with $d_g = 567 \pm 823$ nm and $h_g = 44 \pm 49$ nm and d) provides the last coating with $d_g = 859 \pm 1167$ nm and $h_g = 63 \pm 73$ nm. These coating were used for the generation of the third harmonic. Note that they are not uniformly distributed but have rather a grain like structure. The thicknesses are estimated to be 10 nm, 27 nm, 45 nm and 60 nm for a) to d), respectively.

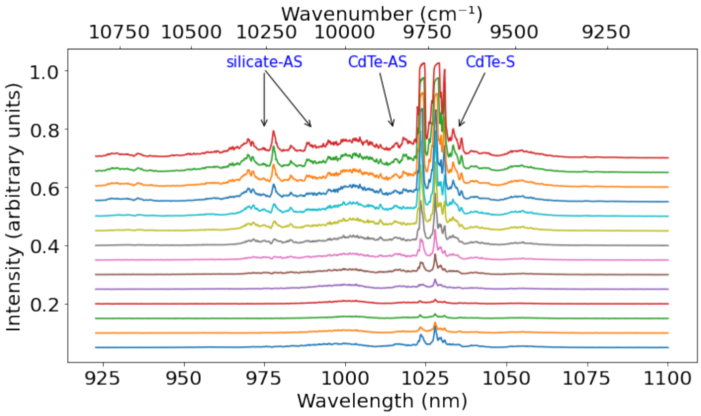

Fig. S8: The Raman spectrum of the fundamental beam reveals the characteristic modes of the host medium at approximately $450\,\mathrm{cm}^{-1}$ and $607\,\mathrm{cm}^{-1}$, along with the CdTe phonon modes ranging from $120\,\mathrm{cm}^{-1}$ to $170\,\mathrm{cm}^{-1}$. Notably, the Stokes shift is absent for the silicate glass, while both Stokes (S) and Anti-Stokes (AS) shifts are observed for the CdTe modes. The different colors represent variations in pump power. As parameter of the different spectral traces the pulse energy of the fundamental ranging from 30 - 80 μJ, or intensities from (1.6 - 4.5) $\cdot 10^{13}$ W/cm^2 was chosen.

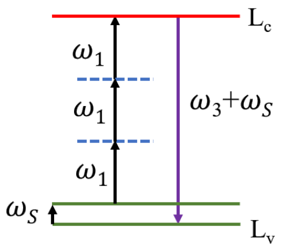

Fig. S9: Schematic description of the hyper-Raman process which generates the blue parts of the harmonic spectra, where ω_s is the Stokes shift.

7 Experimental setup and parameters

The experimental set-up, depicted in Fig. S10, consists of a high-power ytterbium-doped fiber laser (Active Fiber Systems) emitting at a central wavelength of $\lambda = 1030\,\text{nm}$ and $\Delta\lambda = 100\,\text{nm}$. The laser is operated at a repetition rate of $55\,\text{kHz}$, a pulse duration of $\tau = 40\,\text{fs}$, and pulse energies up to $80\,\mu\text{J}$. The beam passes through a polarizing attenuator which separates the horizontally and vertically polarized components from which the intensity of the radiation can be controlled. The beam is then focused onto the target using an almost dispersionless ultra-thin fused silica lens with a focal length of $f = 150\,\text{mm}$, resulting at peak intensities of $5 \cdot 10^{13}\text{W/cm}^2$ at the target surface for a focal spot size of $A_{spot} \approx 40\,\mu\text{m}^2$ (FWHM). The CdTe QDs are coated on the backside of the substrate as shown in Fig. S10 (Target). The generated radiation propagates towards another UV fused silica lens with a focal length of $f = 150\,\text{mm}$. This lens is used to collimate the genrated UV beam. To separate the fundamental beam from the generated harmonics, two calcium fluoride (CaF_2) prisms are positioned in the beam path. The prisms are aligned to minimize the deflection angle. The third harmonic is directed towards an Avantes spectrometer with a grating of 2400 lines/mm, while the fifth harmonic is focused onto the mentioned spectrometer slit and a grating of 3600 lines/mm.

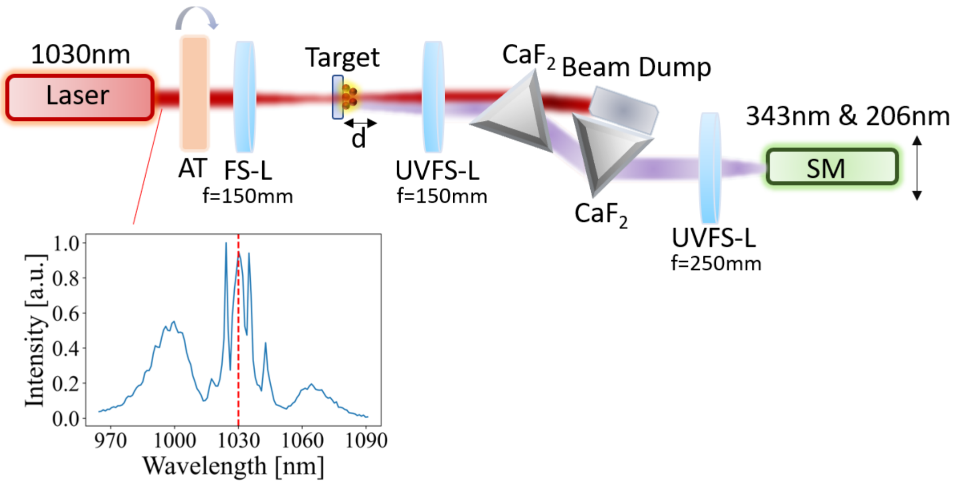

Fig. S10: Schematic drawing of the experimental set-up. The pulse energy of the laser is controlled by an ultra thin polarizing attenuator(AT). Afterwards the pulse propagates towards a $2f$ system in which the target is placed at the focal spot. The harmonics are separated by a CaF_2 prism pair and the fundamental propagates towards the beam dump (BD). The third and fifth harmonic are focused onto a spectrometer (SM). The laser emission spectrum shown below is centered at $\lambda = 1030\,\text{nm}$.

8 Experimental results

8.1 Harmonics in borosilicate glass target

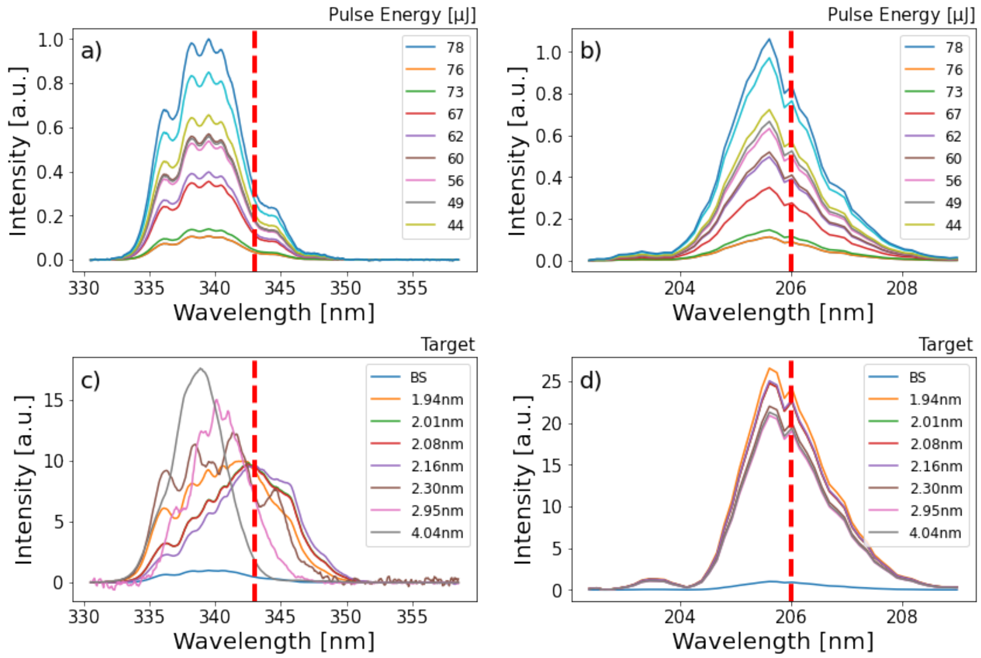

Fig. S11: a) and b) display the power dependent 3rd and 5th harmonic generated in a pure borosilicate glass target, respectively. The red line indicates the integer value for the third (343 nm) and fifth (206 nm) harmonic, respectively. c), d) display the harmonic spectra with CdTe quantum dots coated on the substrate. The highest harmonic yield is depicted for each dot size, which was obtained for a film thickness of 45 nm.

In Fig. S11 a) the yield of the third harmonic is presented for pulse energies ranging from 40 to 80 μJ using a pure borosilicate glass target. The harmonic yield is normalized to the maximum value. In the case of the uncoated glass target, several important features can be observed. First, the maximum of the harmonic is blue-shifted from the position of 343 nm expected for the 3rd harmonic of the maximum of the fundamental radiation ($\lambda_1 = 1030$ nm). Further, the integer harmonic at 343 nm is strongly suppressed, indicated by a sharp drop in the yield. Additionally, several other peaks are visible in the harmonic spectrum at wavelengths of $\lambda = 336$ nm, 338 nm, 339 nm, and 341 nm. These additional peaks can be attributed to Raman lines (see Tab. S3 in the supplemental material) originating from the amorphous silica in the borosilicate glass. The shift of the 343 nm harmonic to 336 nm can be associated with the 607 cm^{-1} Raman line which can be assigned to a defect or structural anomalies in SiO_4 [16]. The shift to 338 nm is related to the 450 cm^{-1} line corresponding to the bending vibration mode of the Si-O-Si and Si-O-B bonds [2, 16]. The appearance of the TA mode at 130 cm^{-1} gives rise to the shift to 341 nm and the edge at 345 nm. Finally, the shift to 339 nm can be attributed to the LA mode at 354 cm^{-1}. These observations highlight the influence of the glass composition and its Raman and phonon modes on the spectral shape and position of the third harmonic. In Fig. S11 c), the third harmonic from the CdTe QD films of about 45 nm thickness shows intensities 10 to 17 times larger than that of the pure borosilicate substrate alone. Further, the spectra differ very strongly from that of the borosilicate glass target. A detailed description follows in the next section.

In Fig. S11 b), the fifth harmonic of borosilicate alone is shown which appears at 206 nm as expected. As indicated in Tab. S3 in the supplemental material some shoulders around this peak can

be assigned to the Raman lines of amorphous silica in the borosilicate glass. Another small peak is observed at 203.5 nm, which is also in close proximity to the resonant peaks and corresponds to the 607 cm^{-1} Raman line. However, it is noteworthy that this additional peak is relatively weak, indicating that the Raman and phonon shifts do not play a significant role in the fifth harmonic generation compared to the third harmonic. Fig.S11 d) shows the fifth harmonic with QDs and no difference except a much higher yield by about a factor of 25 in the peak is observed. Thus, the fifth harmonic remains almost unaffected while the third harmonic is strongly influenced by the presence of the QDs.

9 Spectra generated in other QDs not shown in the main text

The spectra shown below belong to the QD diameters ($d = 2.01 - 2.95$ nm) between the largest and the smallest dots. The red line indicates the resonant position at $\lambda_3 = 343$ nm. The spectra for the small dots (Fig. S12 - S15) are characterized by the appearance of clearly visible sub-peaks which vanish for the larger dots (Fig. S16) as described earlier. The modulation of the resonant third harmonic is present for the dots between $d = 2.01$ nm and $d = 2.16$ nm (Fig. S12 - S14) for one particular thickness $d = 45$ nm. The QD with intermediate diameter (Fig. S15) show this modulation, but not as clearly as for the previous mentioned small dots. The modulation clearly vanishes for the larger dot of diameter $d = 3.94$ nm (Fig. S16). Also the appearance of the sub-peaks (coupling to phonons) decreases with increasing size, which matches the literature[3, 4, 7, 19].

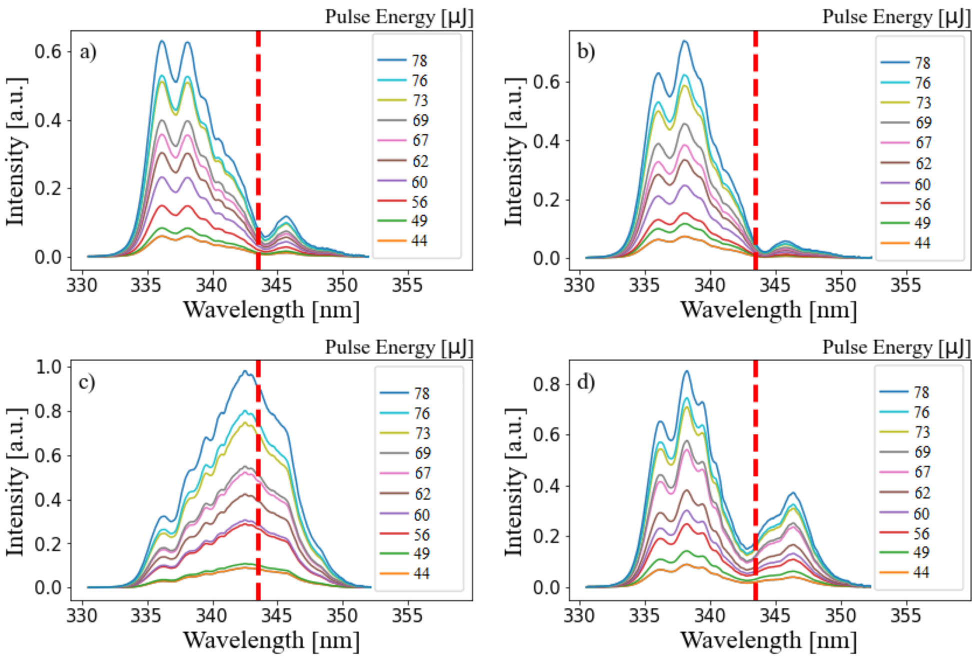

Fig. S12: Generated 3rd harmonic yield for CdTe with a size of 2.01 nm and for film thickness of a) 10 nm, b) 27 nm, c) 45 nm and d) 60 nm.

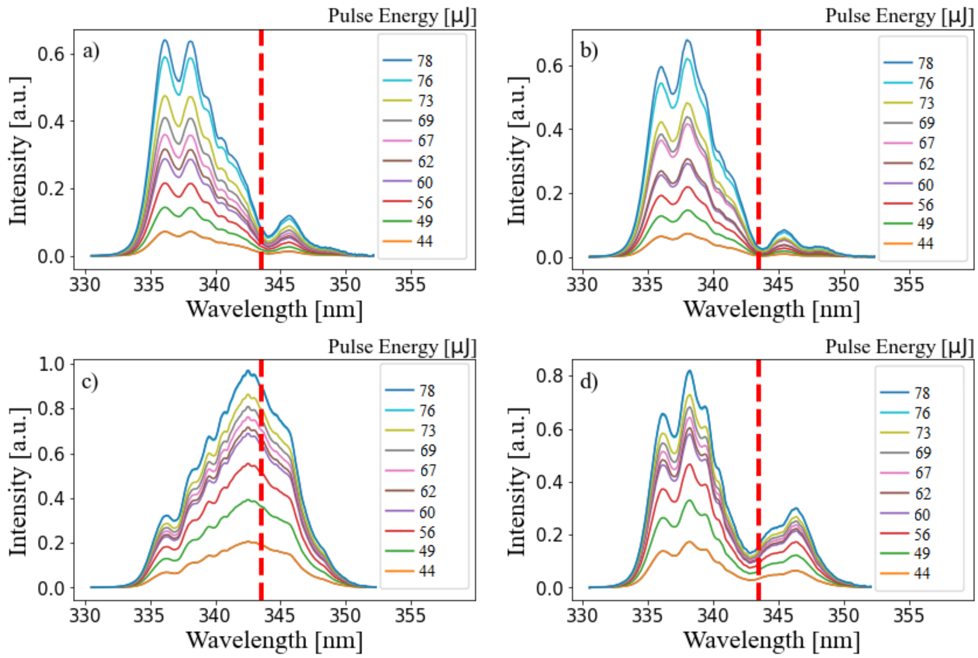

Fig. S13: Generated 3rd harmonic yield for CdTe with a size of 2.08 nm and for film thickness of a) 10 nm, b) 27 nm, c) 45 nm and d) 60 nm.

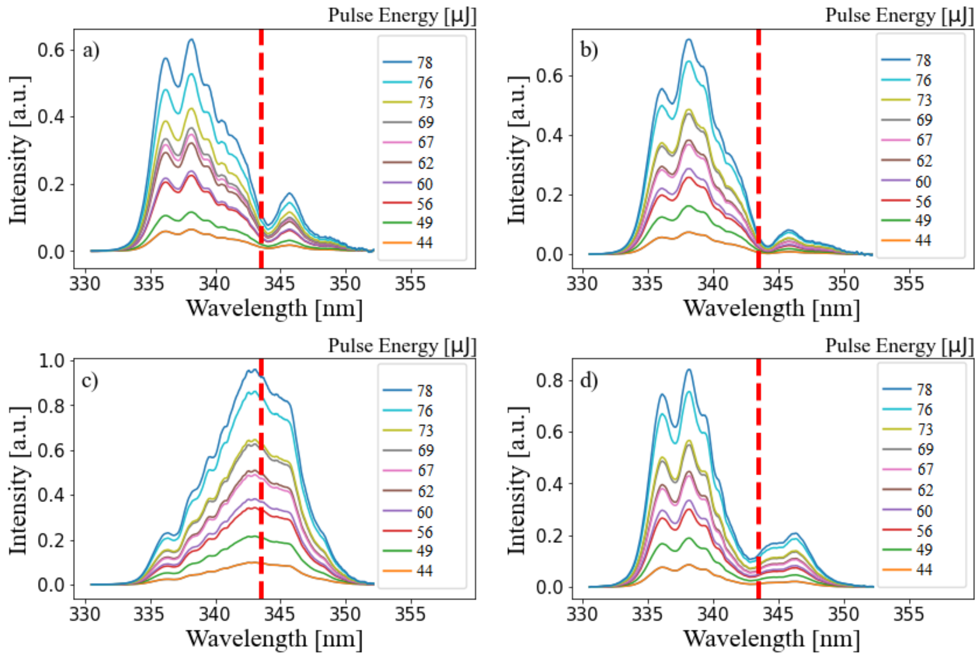

Fig. S14: Generated 3rd harmonic yield for CdTe with a size of 2.16 nm and for film thickness of a) 10 nm, b) 27 nm, c) 45 nm and d) 60 nm.

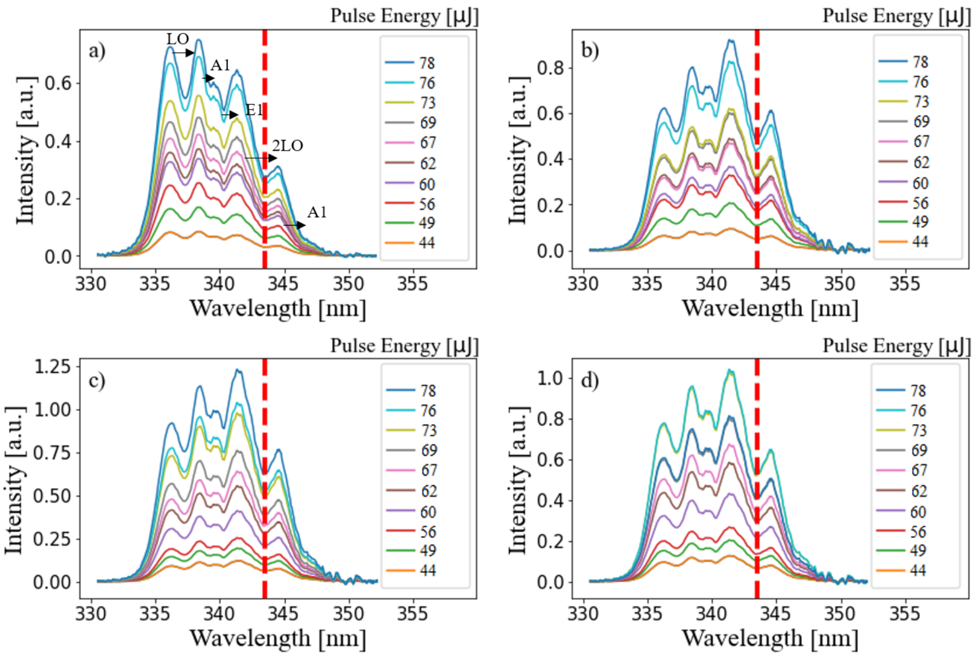

Fig. S15: Generated 3rd harmonic yield for CdTe with a size of 2.30 nm and for film thickness of a) 10 nm, b) 27 nm, c) 45 nm and d) 60 nm. In addition the shift of the peaks according to the LO and TO modes are indicated.

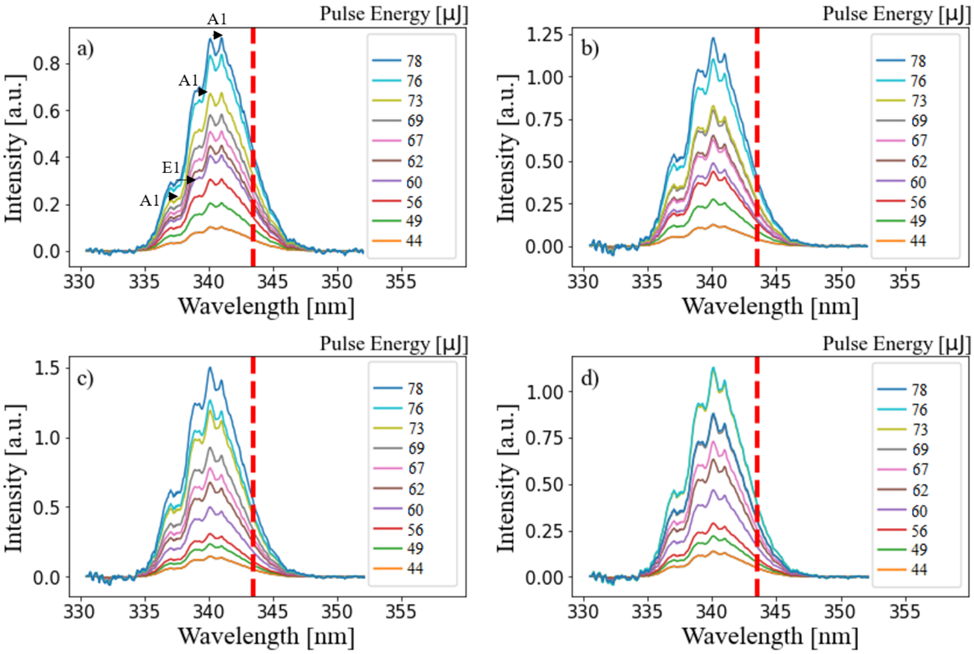

Fig. S16: Generated 3rd harmonic yield for CdTe with a size of 2.95 nm and for film thickness of a) 10 nm, b) 27 nm, c) 45 nm and d) 60 nm. The coupling to the LO modes of CdTe disappeared completely.

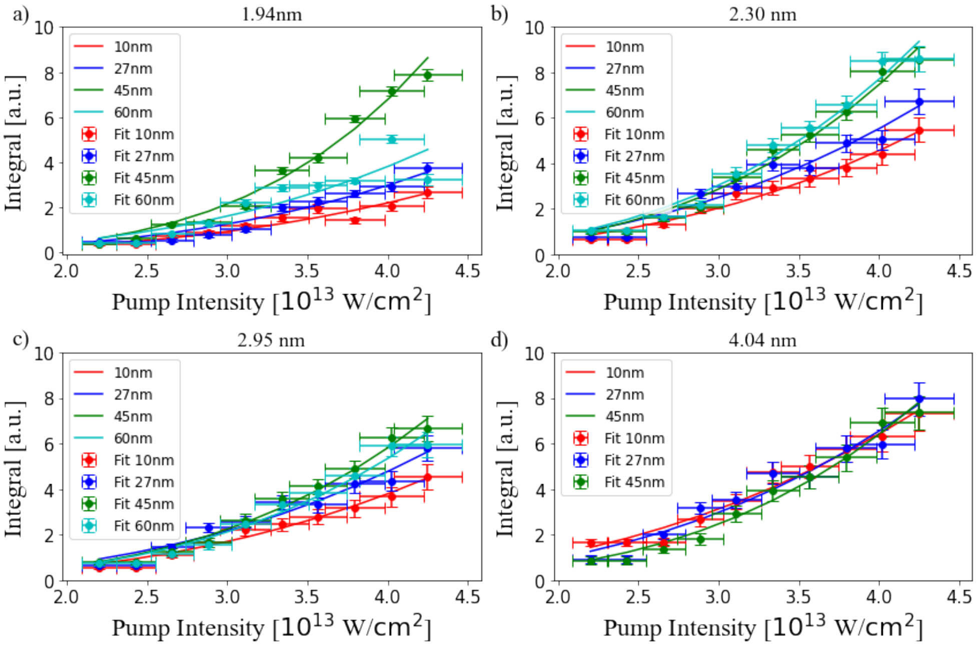

Fig. S17: Spectral yield for the coated thicknesses with CdTe Quantum dots with a size of a) 1.94 nm b) 2.30 nm, c) 2.95 nm and d) 4.04 nm. For QDs with diameter 1.94 nm the dependence on pump energy for a thickness of 45 nm varies with $I^{3.5}$, and the other three thicknesses with I^3. The situation is similar for the QDs with sizes 2.01 nm, 2.08 nm and 2.16 nm. The other yields depicted in b) to c) vary with I^3 for all coated thicknesses.

9.1 Harmonic yield

In this section we will analyze the dependence of the harmonic yield of the third harmonic on the incident pump intensity for different quantum dot sizes and film thicknesses. This yield is depicted in Fig. S17 a) - d) for the sizes of a) 1.94 nm b) 2.30 nm, c) 2.95 nm and d) 4.04 nm, respectively. For this purpose the entire spectral integral is taken into account due to the presence of spectral broadening. In a), for the film thickness of 45 nm the dependence on pump intensity results in $Y \propto I^{3.5}$, and for the other three thicknesses to about $Y \propto I^3$. The situation is similar for the QDs with sizes 2.01 nm, 2.08 nm and 2.16 nm which are not shown here. The other yields depicted in Fig. S17 b) to c) are proportional to the third power of the fundamental intensity I^3 for all coated thicknesses.

Fig. S18 shows the trend of the third order susceptibility according to eq. (S45) as a function of the size of the QDs for a film thickness of 45 nm (red graph) and of 10 nm (green graph). The behaviour of the nonlinear optical response can be described by resonant and non-resonant contributions to the $\chi^{(3)}$ by free electrons of the conduction bands according to eq. (S41), which also matches the nonlinear optical response measured in nanosized metal particles before[8, 18]. For a thickness of 45 nm the the susceptibility slightly decreases with increasing QD size d and increases afterwards. For a thickness of 10 nm the decrease for the smaller dots is absent and the yield increases constantly with increasing size indicating a weaker influence of the resonant transition described by $\gamma_2\, d^{-3}$ for this particular thickness. Thus, the intraband mechanisms of electrons in the conduction band play a dominant role in the generation of the third harmonic in CdTe QDs. This clear size dependence of resonant and non-resonant contributions to the non-linear susceptibility is crucial to understand the previous measured spectra. The resonant contributions are dominant for smaller dots since $\chi_{res} \propto d^{-3}$, and the non-resonant contributions are more dominant for the larger QDs since $\chi_{non-res} \propto d^2$.

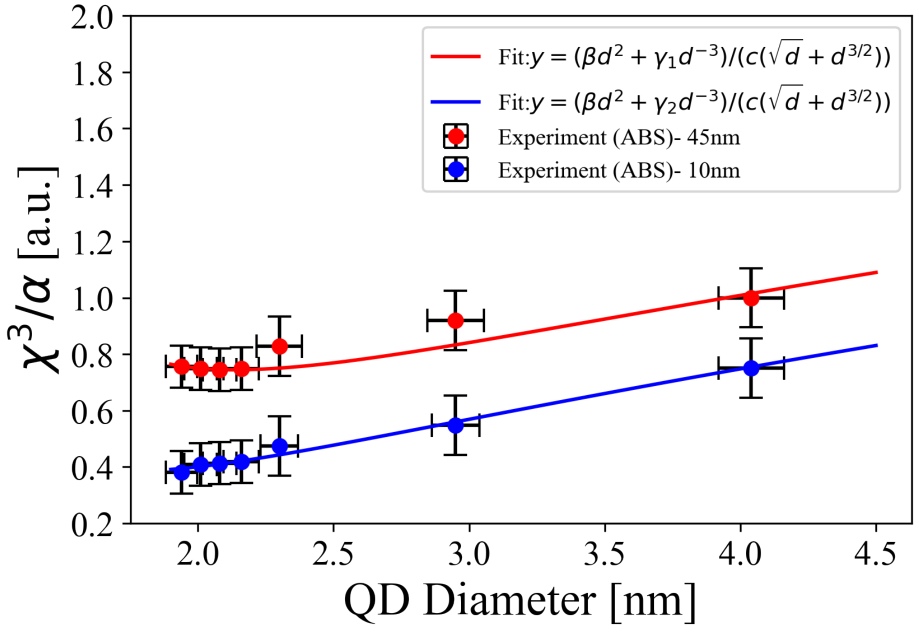

Fig. S18: Size dependent trend of the normalized third order susceptibility according to eq(S45) [8, 14, 15]. The nonlinear optical response $\chi^{(3)}$ shows a $(\beta d^{-3}+\gamma_{1,2}d^2)$-dependence modulated with the one- and three-photon absorption coefficient $\alpha(\omega)$ with respect to the dot diameter d measured with the ABS method for film thickness of 10 nm (blue) and of 45 nm (red). The experimental values are represented by the the square root of the normalized measured intensity. The parameters fitted to the data are $\beta = 0.79 \pm 0.02$, $\gamma_1 = 6.72 \pm 0.05$, $\gamma_2 = 2.81 \pm 0.05$ and $c = 1.25 \pm 0.03$. The blue graph is shifted by 0.2 downwards for better visualization.

9.2 Power dependent yield of the resonant harmonic

In this subsection, we determine the resonant non-linear third-order susceptibility, $\chi^{(3)}_{eff}$, by fitting an I^3 dependence to the resonant harmonic gain obtained by eq. S34. Fig. S19 illustrates the resonant harmonic gain for all QD sizes and film thicknesses, normalized to the maximum value obtained for the smallest QDs, as shown in panel (a). It is immediately evident that QDs with sizes larger than 3 nm (Fig. S19 c) and d) exhibit no significant thickness-dependent gain in the resonant third harmonic. In contrast, this behavior is consistently observed for QDs ≤ 2.30 nm. Therefore, a film thickness-dependent modulation is exclusively present in smaller QDs.

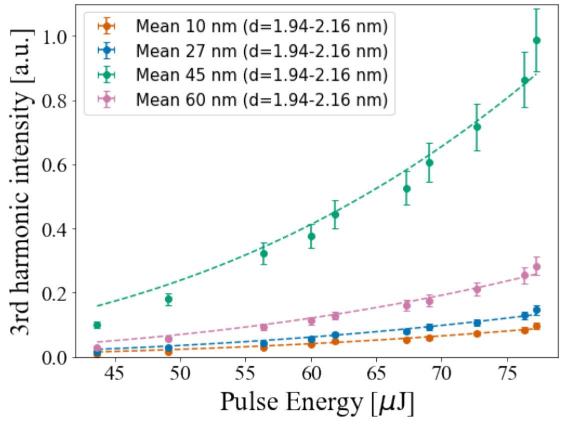

(a) Intensity of 343 nm of QDs with sizes $1.94 - 2.16$ nm for all thicknesses as a function of pulse energy.

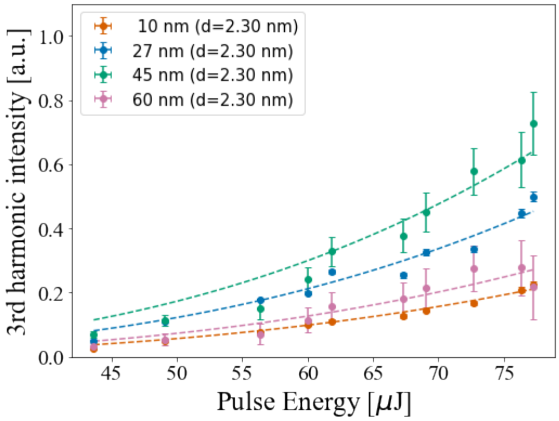

(b) Intensity of 343 nm for the QDs with size 2.3 nm for all thicknesses as a function of pulse energy.

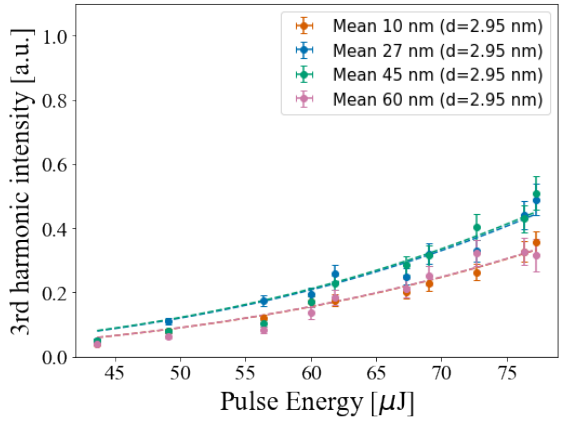

(c) Intensity of 343 nm for the QDs with size 2.95 nm for all thicknesses as a function of pulse energy.

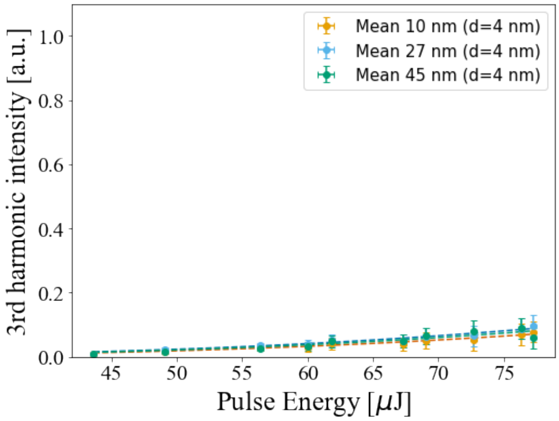

(d) Intensity of 343 nm for the QDs with size 4.04 nm for all thicknesses as a function of pulse energy.

Fig. S19: Comparison of 343 nm intensities for QDs of different sizes as a function of pulse energy. The dashed lines correspond to an I^3 dependent yield.

10 Fluctuations in intensity - Determination of the uncertainty

The deviations of the intensity of the harmonic yield (error bars in Fig. S17) are gained by a time dependent measurement. The harmonic spectra were measured over 3 hours for a pulse energy of $E_{\text{pulse}} = 80\,\mu\text{J}$ ($I = 5 \cdot 10^{13}\,\frac{\text{W}}{\text{cm}^2}$) and the fluctuations of the intensity which are about 13% are displayed in Fig. S20.

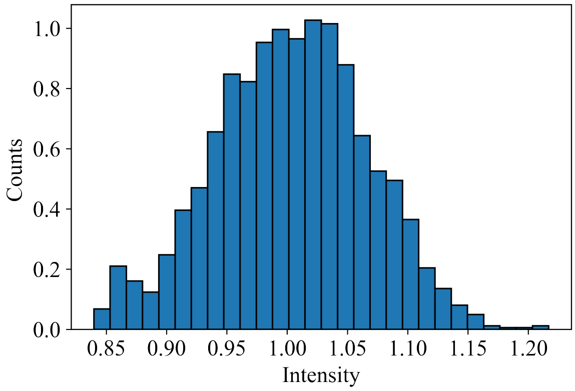

Fig. S20: Histogram for the yield fluctuations of the spectral integral for the third harmonic. The histogram displays fluctuation of about 13%.

11 DFT band structure

The band structure of bulk CdTe was calculated using the framework octopus [20–23]. The ground state calculation was performed using the local density approximation (LDA) with the Hartwigsen-Goedecker-Hutter (hgh) pseudo-potential set as an approximation of the energy exchange correlation functional (XC) [24]. We employed a 6x6x6 Monkhorst-Pack grid with four shifts to get sufficient convergence. We calculated the Brillouin zone along the $\overline{\Gamma\text{L}}$, $\overline{\Gamma\text{X}}$, and $\overline{\text{X}\Gamma'}$ using a sampling of 10-10-15 k-points.

References

[1] B. Champagnon, G. Panczer, C. Chemarin, B. Humbert-Labeaumaz, "Raman study of quartz amorphization by shock pressure," *J. Non-Cryst. Solids*, vol. 196, pp. 221-226, 1996.

[2] M. H. Manghnani, A. Hushur, T. Sekine, J. Wu, J.F. Stebbins, Q. Williams, "Raman, Brillouin, and nuclear magnetic resonance spectroscopic studies on shocked borosilicate glass," *J. Appl. Phys.*, vol. 109, p. 113509, 2009.

[3] X.-Q. Li , H. Nakayama, and Y. Arakawa, "Lifetime of Confined LO Phonons in Quantum Dots and Its Impact on Phonon Bottleneck Issue," *Jpn. J. Appl. Phys.*, vol. 38, p. 473, 1999.

[4] G. Morello, M. De Giorgi, S. Kudera, L. Manna, R. Cingolani, and M. Anni, "Temperature and Size Dependence of Nonradiative Relaxation and Exciton-Phonon Coupling in Colloidal CdTe Quantum Dots," *J. Phys. Chem C.*, vol. 111, pp. 5846–5849, 2007.

[5] T. Rajh, O. I. Micic, and A. J. Nozik, "Synthesis and characterization of surface-modified colloidal CdTe quantum dots," *J. Phys. Chem.*, vol. 97, 46, pp. 11999–12003, 1993.

[6] A. Efros, Al. L. Efros, "Interband Light Absorption in Semiconductor Spheres," *Sov. Phys. Semicond.*, vol. 16, pp. 772-775, 1982.

[7] F. de Moure-Flores, J.G. Quiñones-Galván, A. Guillén-Cervantes et al., "CdTe thin films grown by pulsed laser deposition using powder as target: Effect of substrate temperature," *J. Cryst. Growth*, vol. 386, pp. 27–31, 2014.

[8] V. P. Drachev, A. K. Buin, H. Nakotte, V. M. Shalaev, "Size dependent χ^3 for Conduction Electrons in Ag Nanoparticles," *Nano Lett.*, vol. 4, pp. 1535-1539, 2014.

[9] F. Hache, D. Ricard, C. Flytzanis, "Optical nonlinearities of small metal particles surface mediated resonance and quantum size effects," *J. Opt. Soc. Am. B*, vol. 3, pp. 1647-1655, 1986.

[10] D. Ricard, P. Roussignol, and C. Flytzanis, "Surface-mediated enhancement of optical phase conjugation in metal colloids," *Opt. Lett.*, vol. 10, pp. 511-513, 1985.

[11] I. Moreels, K. Lambert, D. Smeets et al., "Size-Dependent Optical Properties of Colloidal PbS Quantum Dots," *ACS Nano*, vol. 3, pp. 3023–3030, 2009.

[12] D. J. Jackson and J. J. Wynne, "Interference effects between different optical harmonics," *Phys. Rev. Lett.* vol. 49, p. 8, 1982.

[13] D. J. Jackson, J. J. Wynne, and P.H. Kes, "Resonance-enhanced multiphoton ionization interference effects due to harmonic generation," *Phys. Rev. A*, vol. 49, p. 8, 1983.

[14] L. Máthé, C.P. Onyenegecha, A.-A. Farcas, L.-M. Pioras-Ţimbolmas, M. Solaimani, H. Hassanabadi, "Linear and nonlinear optical properties in spherical quantum dots: Inversely quadratic Hellmann potential," *Phys. Lett. A*, vol. 397, p. 127262, 2021.

[15] C.P. Onyenegecha, "Linear and nonlinear optical properties in spherical quantum dots: Modified Möbius squared potential," *Heliyon*, vol. 8, p. e10387, 2022.

[16] B. Champagnon, G. Panczer, C. Chemarin, B. Humbert-Labeaumaz, "Raman study of quartz amorphization by shock pressure," *J. Non-Cryst. Solids*, vol. 196, pp. 221-226, 1996.

[17] M. H. Manghnani, A. Hushur, T. Sekine, J. Wu, J. F. Stebbins, Q. Williams, "Raman, Brillouin, and nuclear magnetic resonance spectroscopic studies on shocked borosilicate glass," *J. Appl. Phys.*, vol. 109, p. 113509, 2009.

[18] K. Ushida, S. Kaneko, S. Omi et al., "Optical nonlinearities of a high concentration of small metal particles dispered in glass: copper and silver particles," *J. Opt. Soc. Am. B*, vol. 11, pp. 1236-1243, 1994.

[19] A. M. Jagtap, J. Khatei, K. S. R. K. Rao, "Exciton phonon scattering and nonradiative relaxation of excited carriers hydrothermally synthesized CdTe quantum dots," *Phys. Chem. Chem. Phys.*, vol. 17, pp. 27579-27587, 2015.

[20] N. Tancogne-Dejean, M. J. T. Oliveira, X. Andrade et al., "Octopus, a computational framework for exploring light-driven phenomena and quantum dynamics in extended and finite systems," *Chem. Phys.*, vol. 152, p. 124119, 2020.

[21] X. Andrade, D. A. Strubbe, U. De Giovannini et al., "Real-space grids and the Octopus code as tools for the development of new simulation approaches for electronic systems," *Phys. Chem. Chem. Phys.*, vol. 17, pp.31371-31396, 2015.

[22] A. Castro, H. Appel, M. Oliveira et al., "*octopus*: a tool for the application of time-dependent density functional theory," *Phys. Stat. Sol.*, vol. 243, pp. 2465-2488, 2006.

[23] M. A. L. Marques, A. Castro, G. F. Bertsch, and A. Rubio, "octopus: a first-principles tool for excited electron-ion dynamics," *Comput. Phys. Commun.*, vol. 151, pp. 60-78, 2003.

[24] C. Hartwigsen, S. Goedecker, J. Hutter, "Relativistic separable dual-space Gaussian pseudopotentials from H to Rn," *Phys. Rev. B.*, vol. 58, p. 3641, 1998.
