# Supplementary figures and images for: Quantum control in size selected semiconductor quantum dot thin films
Source: Nanophotonics. 2025 Jan 16;14(2):229–39. doi: 10.1515/nanoph-2024-0529 (PMC11806504; doi:10.1515/nanoph-2024-0529)

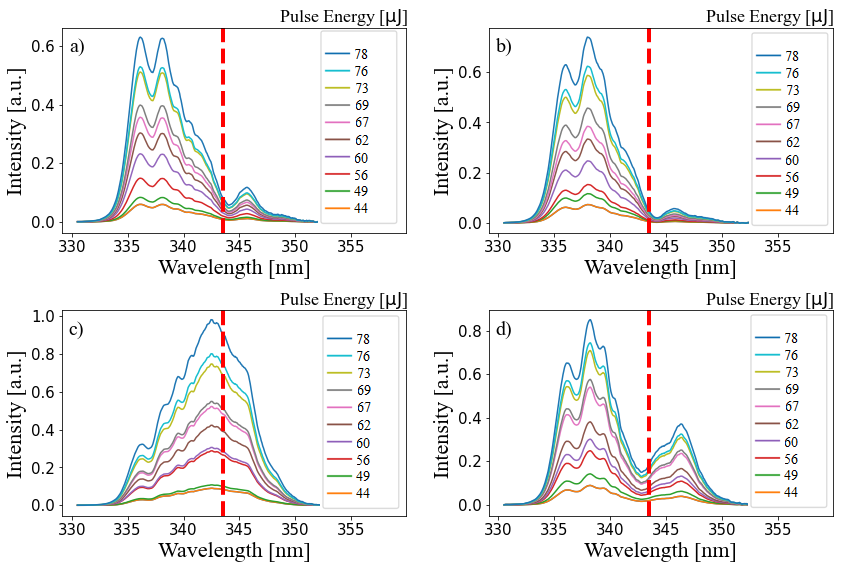

Supplement: Supplementary file 2 — Supplementary Material Details [file j_nanoph-2024-0529_suppl_002.zip › Supplemenatl Revised/1h_3te.png]

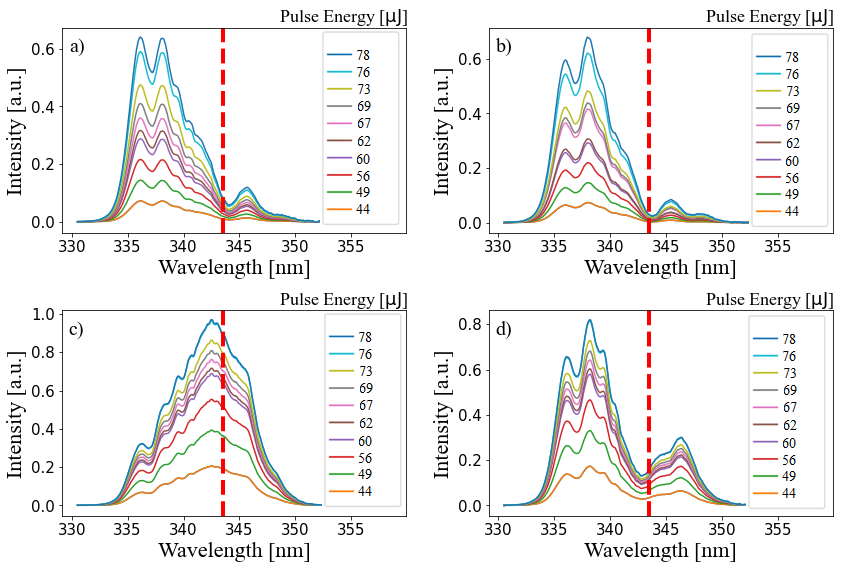

Supplement: Supplementary file 2 — Supplementary Material Details [file j_nanoph-2024-0529_suppl_002.zip › Supplemenatl Revised/2h_3te.png]

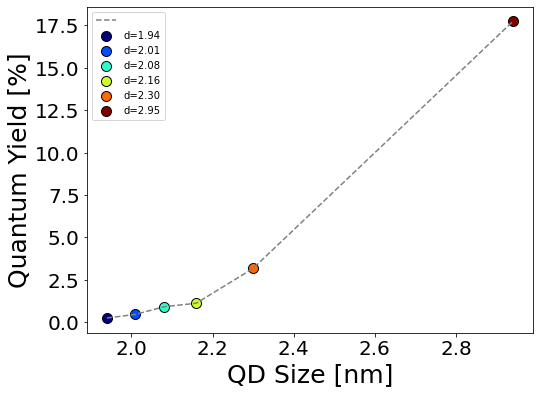

Supplement: Supplementary file 2 — Supplementary Material Details [file j_nanoph-2024-0529_suppl_002.zip › Supplemenatl Revised/QY.png]

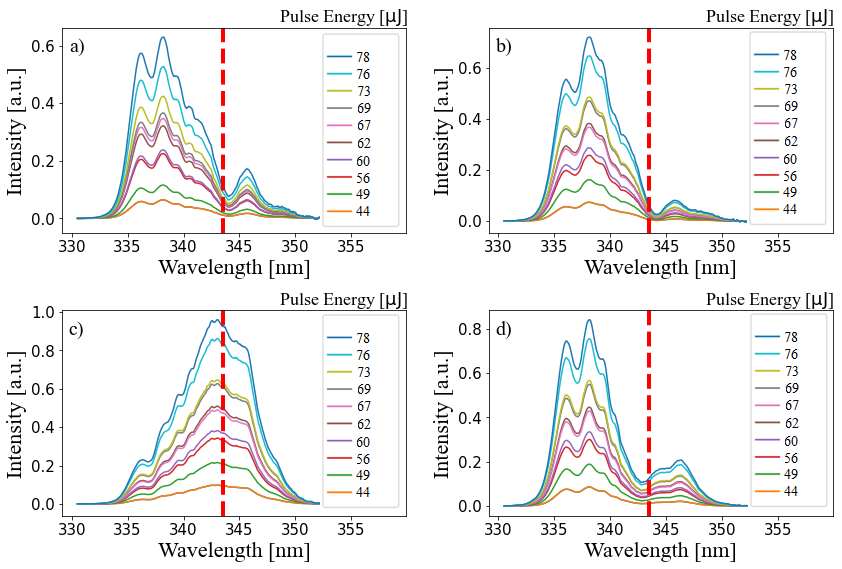

Supplement: Supplementary file 2 — Supplementary Material Details [file j_nanoph-2024-0529_suppl_002.zip › Supplemenatl Revised/4h_3te.png]

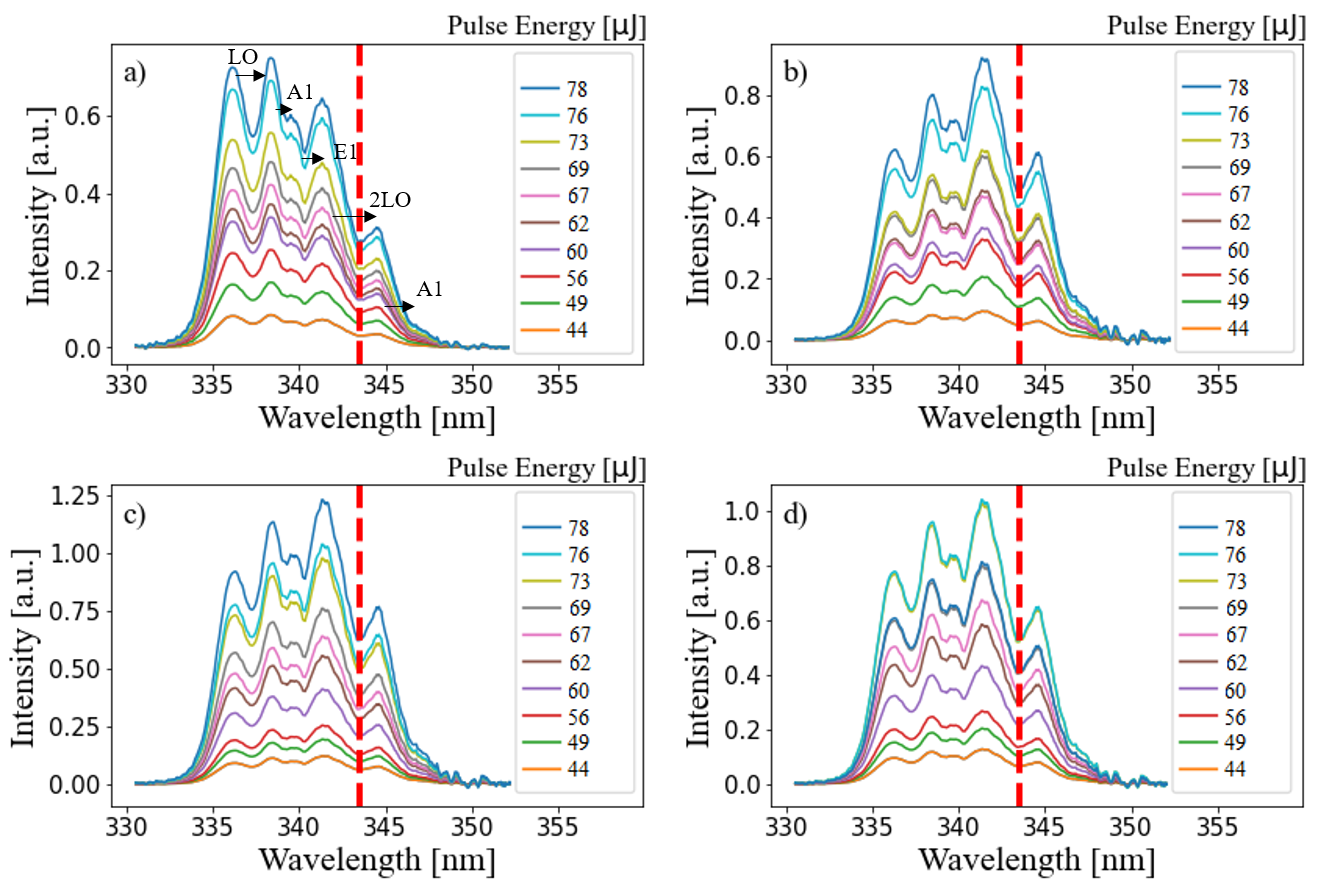

Supplement: Supplementary file 2 — Supplementary Material Details [file j_nanoph-2024-0529_suppl_002.zip › Supplemenatl Revised/6h_3te.png]

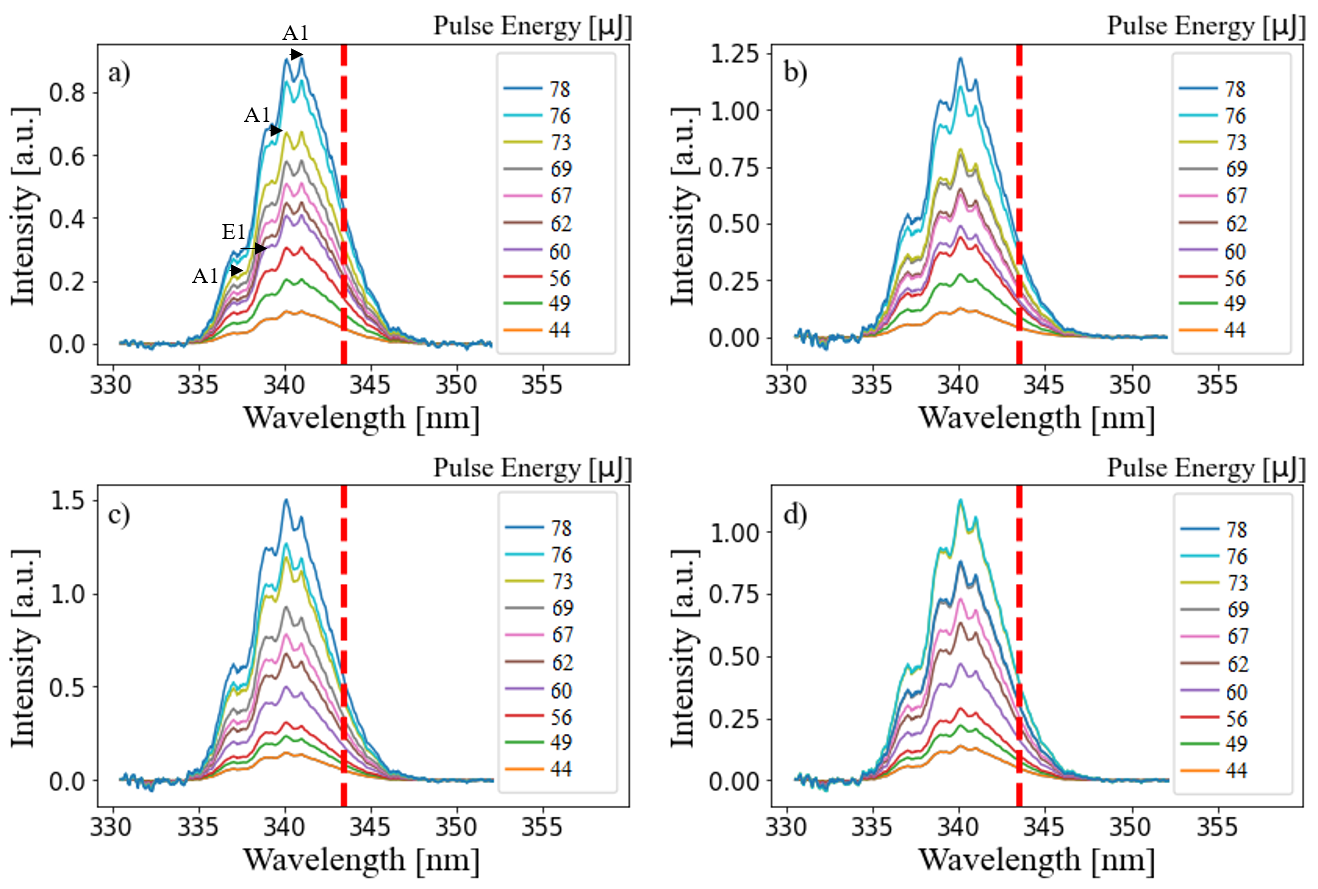

Supplement: Supplementary file 2 — Supplementary Material Details [file j_nanoph-2024-0529_suppl_002.zip › Supplemenatl Revised/24h_3te.png]

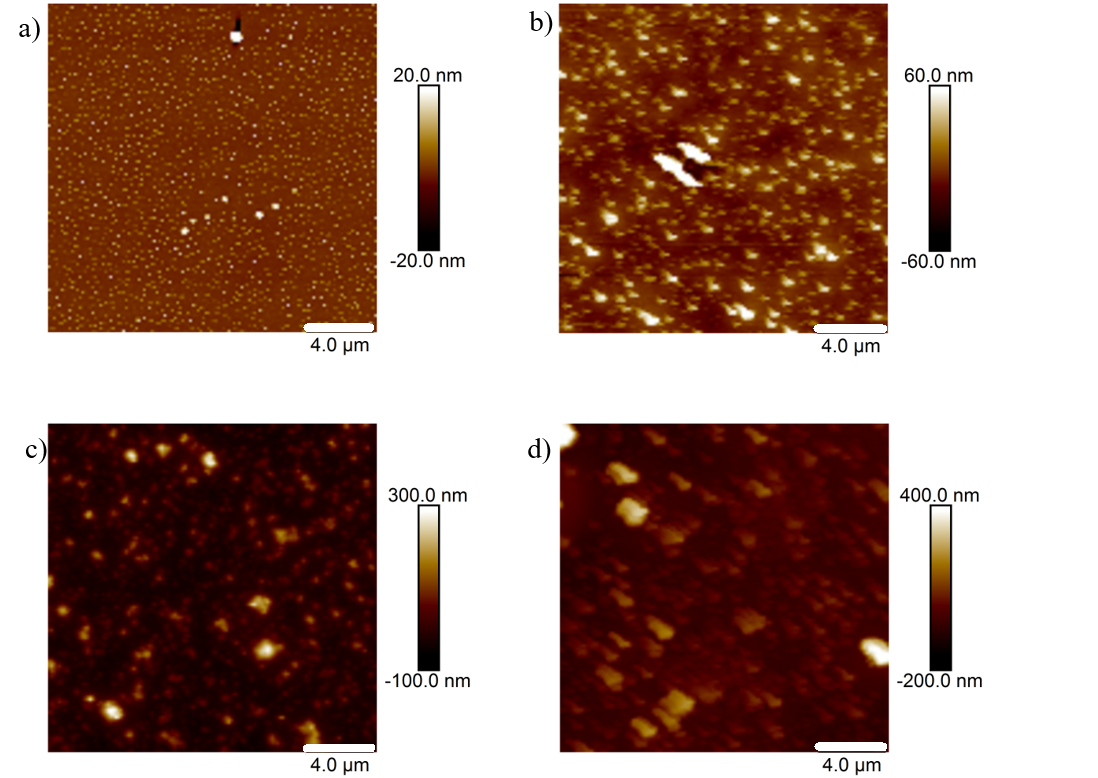

Supplement: Supplementary file 2 — Supplementary Material Details [file j_nanoph-2024-0529_suppl_002.zip › Supplemenatl Revised/24h afm.png]

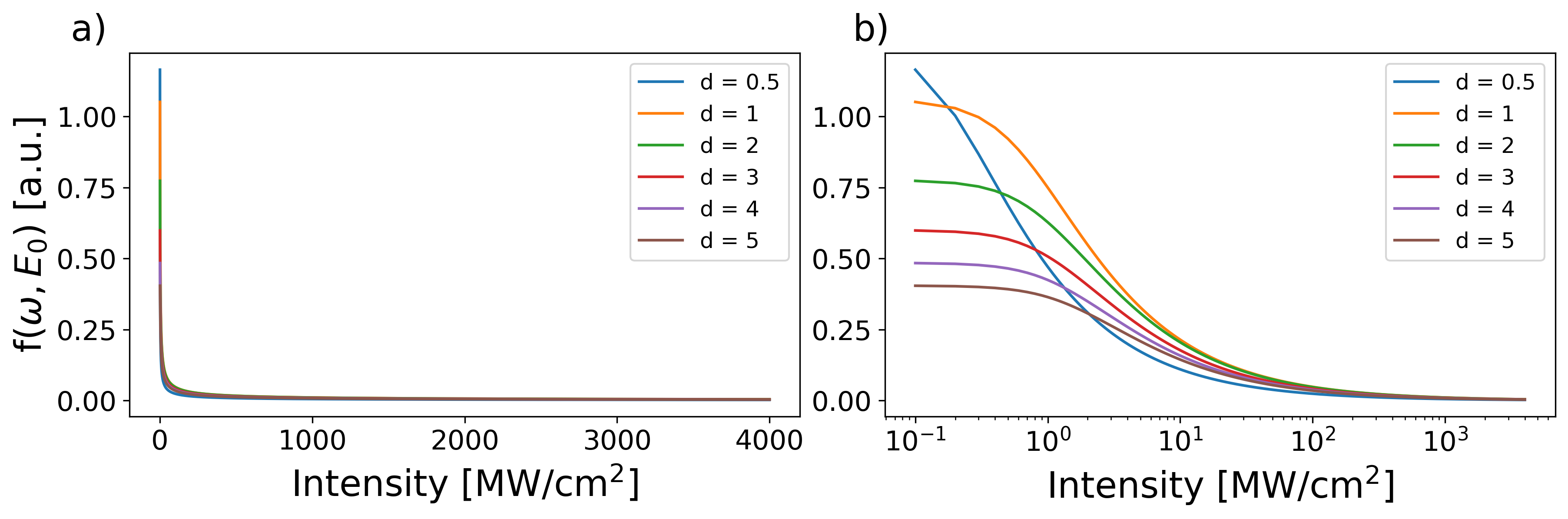

Supplement: Supplementary file 2 — Supplementary Material Details [file j_nanoph-2024-0529_suppl_002.zip › Supplemenatl Revised/FEF.png]

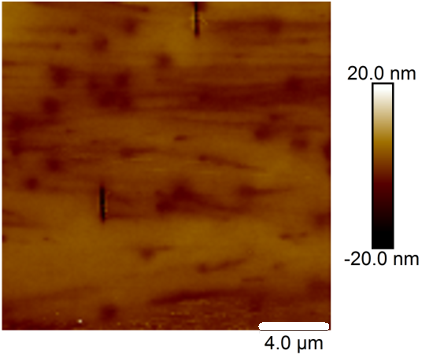

Supplement: Supplementary file 2 — Supplementary Material Details [file j_nanoph-2024-0529_suppl_002.zip › Supplemenatl Revised/FS AFM.png]

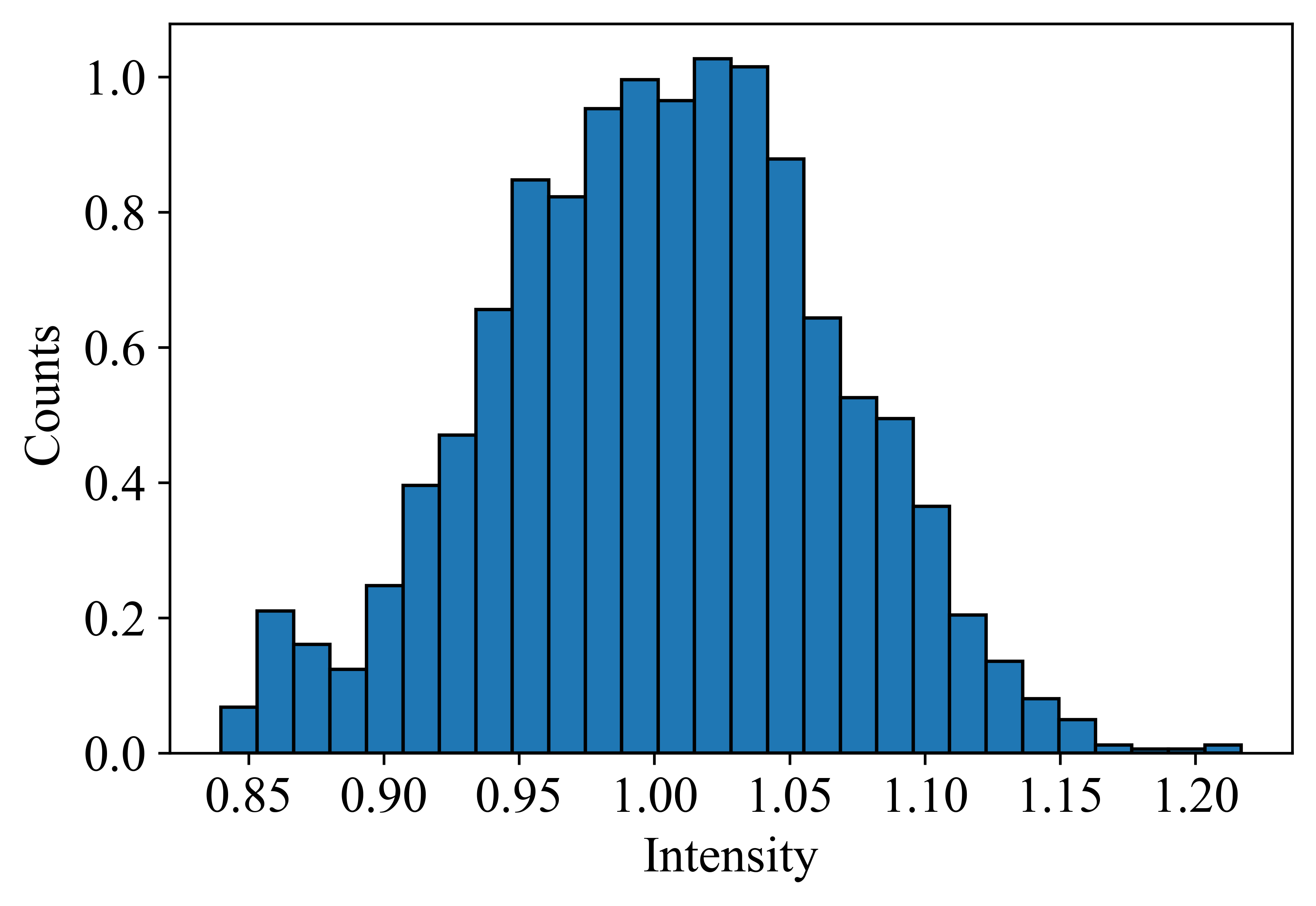

Supplement: Supplementary file 2 — Supplementary Material Details [file j_nanoph-2024-0529_suppl_002.zip › Supplemenatl Revised/HistogrammFehler.png]

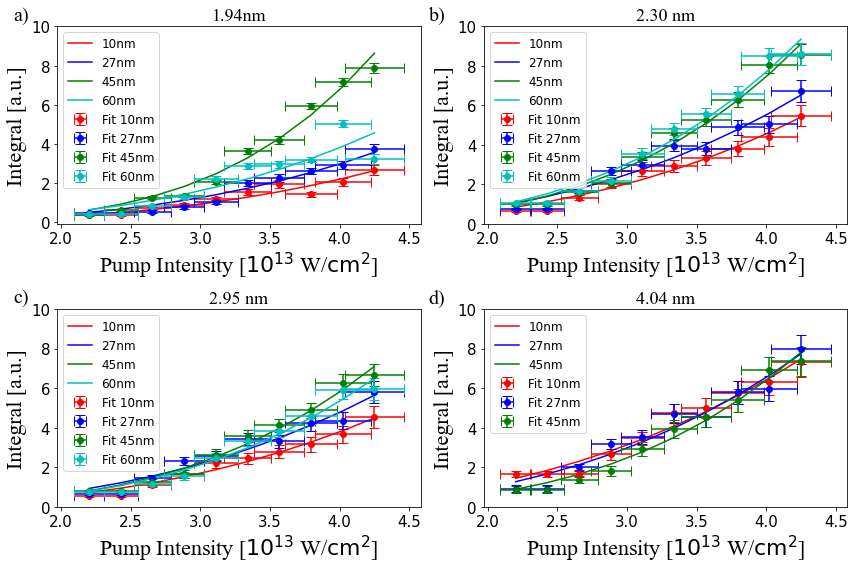

Supplement: Supplementary file 2 — Supplementary Material Details [file j_nanoph-2024-0529_suppl_002.zip › Supplemenatl Revised/P_relevant.png]

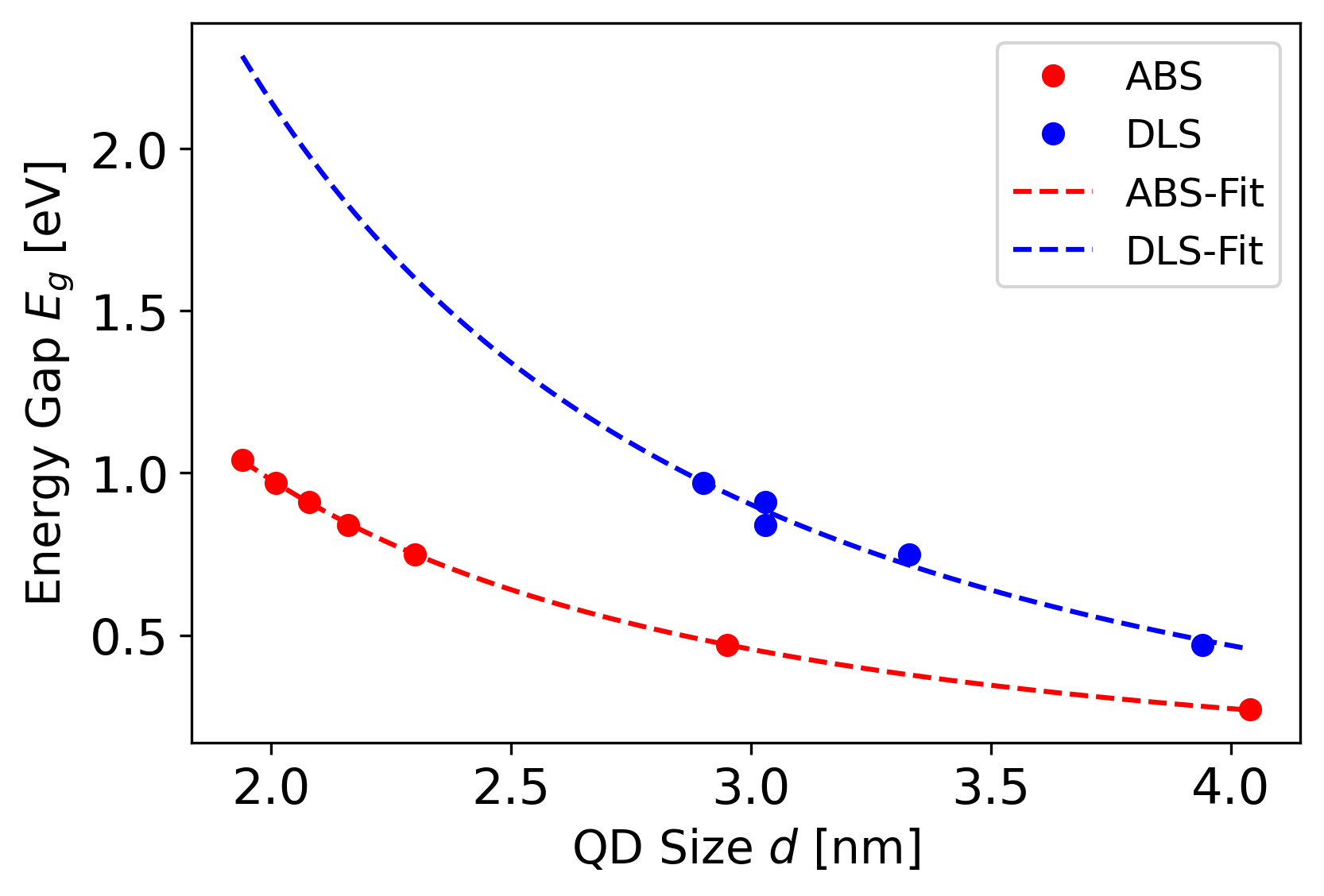

Supplement: Supplementary file 2 — Supplementary Material Details [file j_nanoph-2024-0529_suppl_002.zip › Supplemenatl Revised/QC_Plot.png]

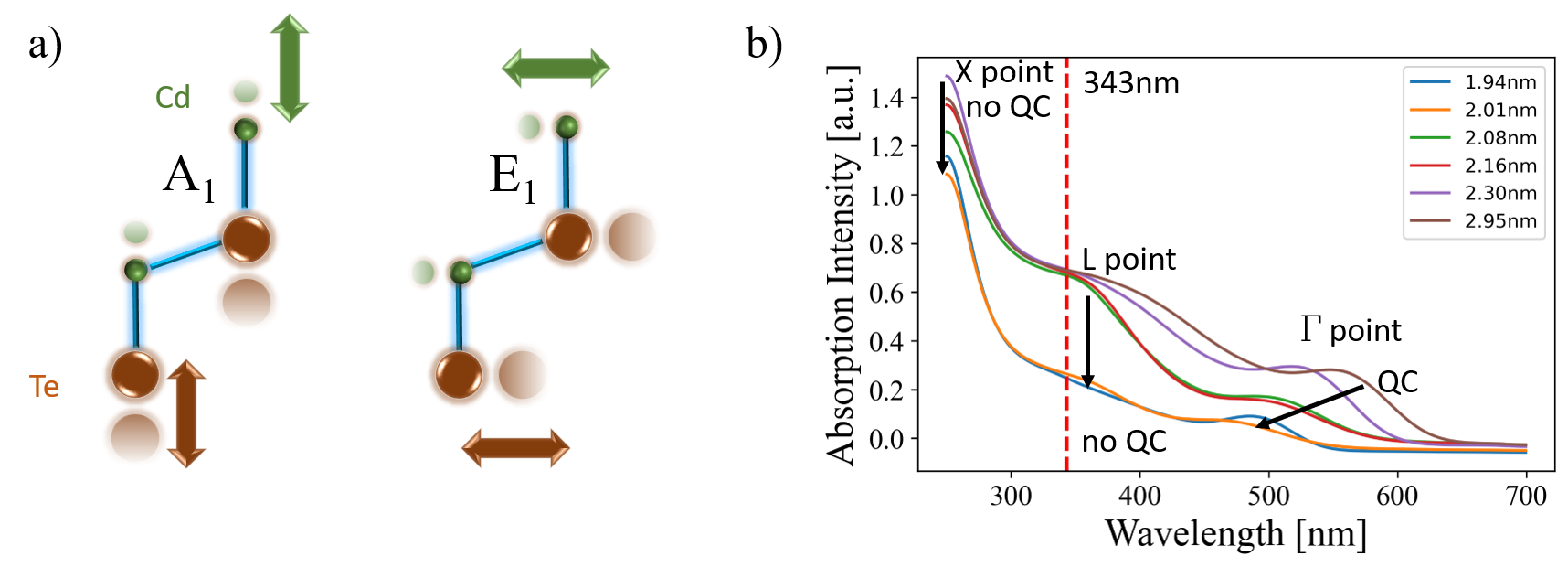

Supplement: Supplementary file 2 — Supplementary Material Details [file j_nanoph-2024-0529_suppl_002.zip › Supplemenatl Revised/qd phonon und absorption.png]

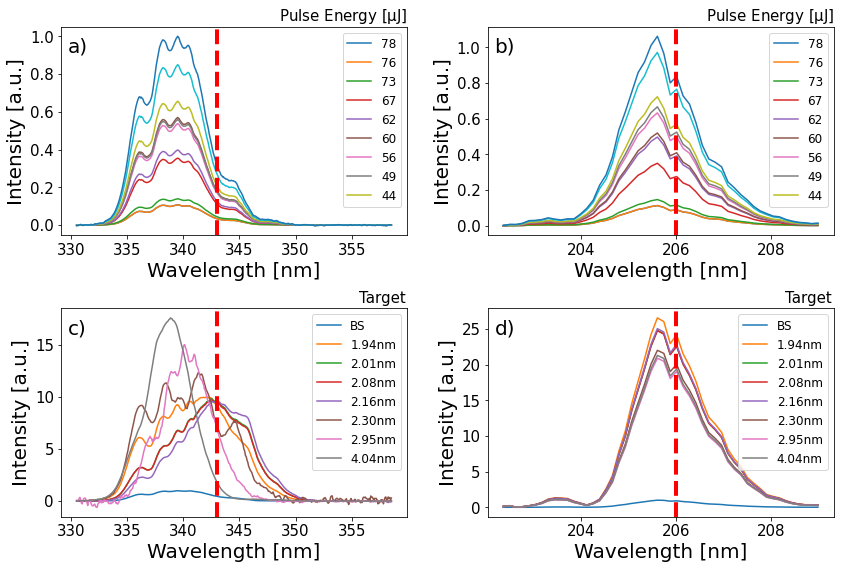

Supplement: Supplementary file 2 — Supplementary Material Details [file j_nanoph-2024-0529_suppl_002.zip › Supplemenatl Revised/Vergleich alle Spektren.png]

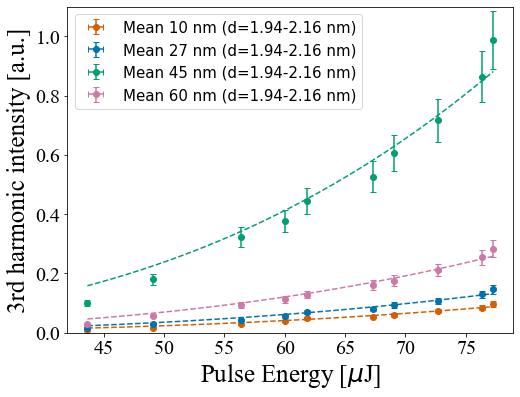

Supplement: Supplementary file 2 — Supplementary Material Details [file j_nanoph-2024-0529_suppl_002.zip › Supplemenatl Revised/3te_dicke.png]

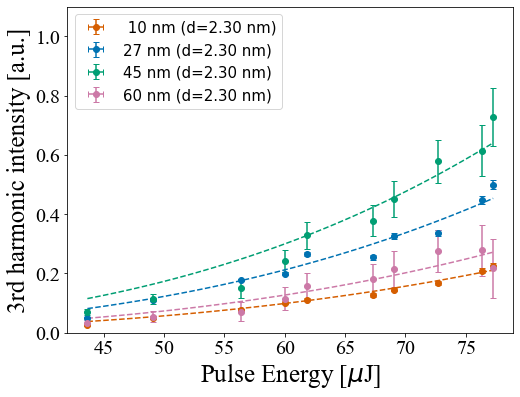

Supplement: Supplementary file 2 — Supplementary Material Details [file j_nanoph-2024-0529_suppl_002.zip › Supplemenatl Revised/3te_dicke230.png]

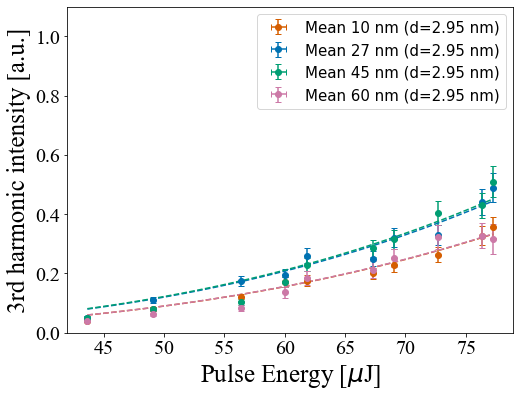

Supplement: Supplementary file 2 — Supplementary Material Details [file j_nanoph-2024-0529_suppl_002.zip › Supplemenatl Revised/3te_dicke295.png]

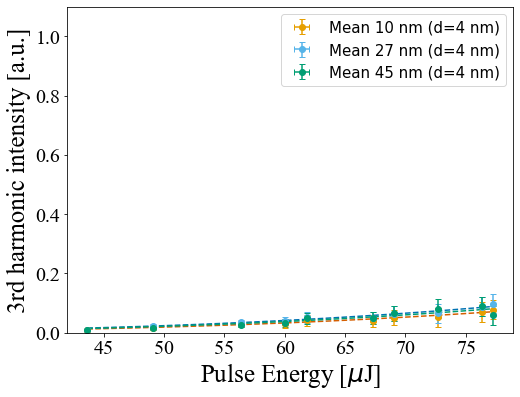

Supplement: Supplementary file 2 — Supplementary Material Details [file j_nanoph-2024-0529_suppl_002.zip › Supplemenatl Revised/3te_dicke404.png]

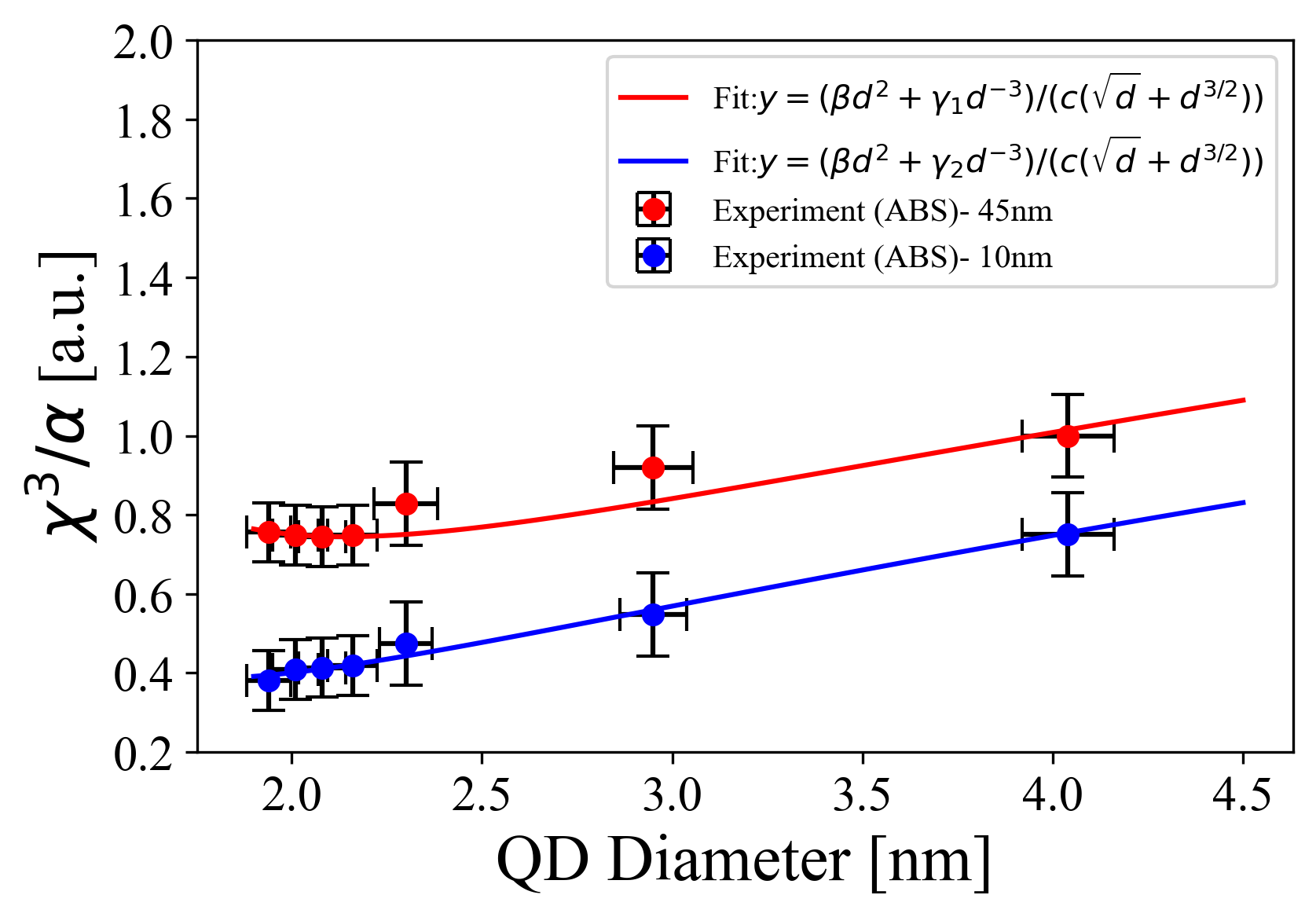

Supplement: Supplementary file 2 — Supplementary Material Details [file j_nanoph-2024-0529_suppl_002.zip › Supplemenatl Revised/3te gesamt.png]

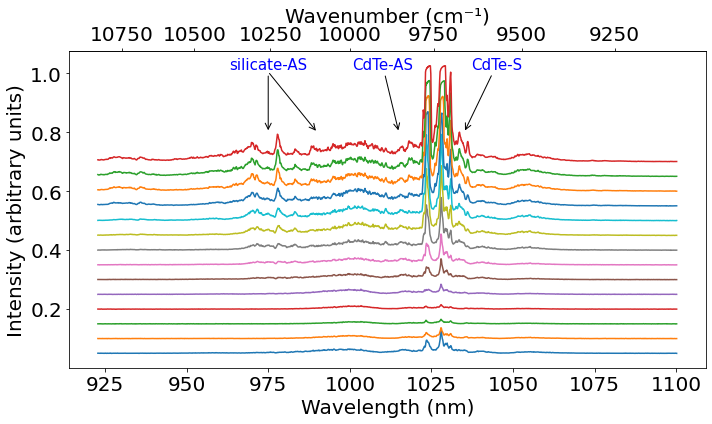

Supplement: Supplementary file 2 — Supplementary Material Details [file j_nanoph-2024-0529_suppl_002.zip › Supplemenatl Revised/raman fund.png]

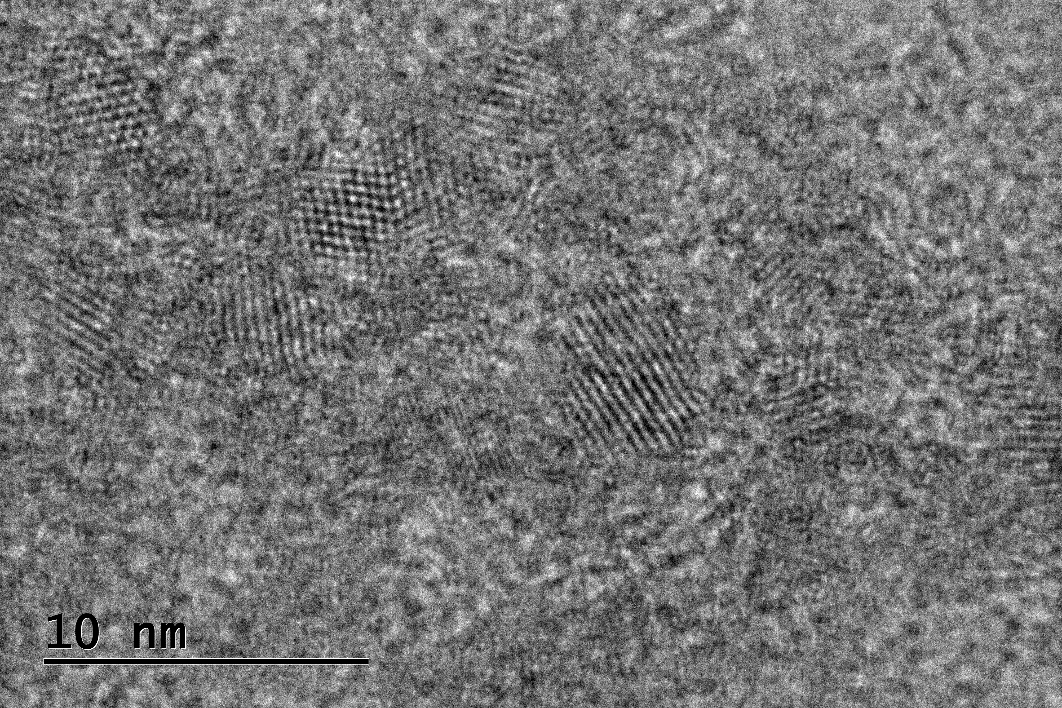

Supplement: Supplementary file 2 — Supplementary Material Details [file j_nanoph-2024-0529_suppl_002.zip › Supplemenatl Revised/TEM1.png]

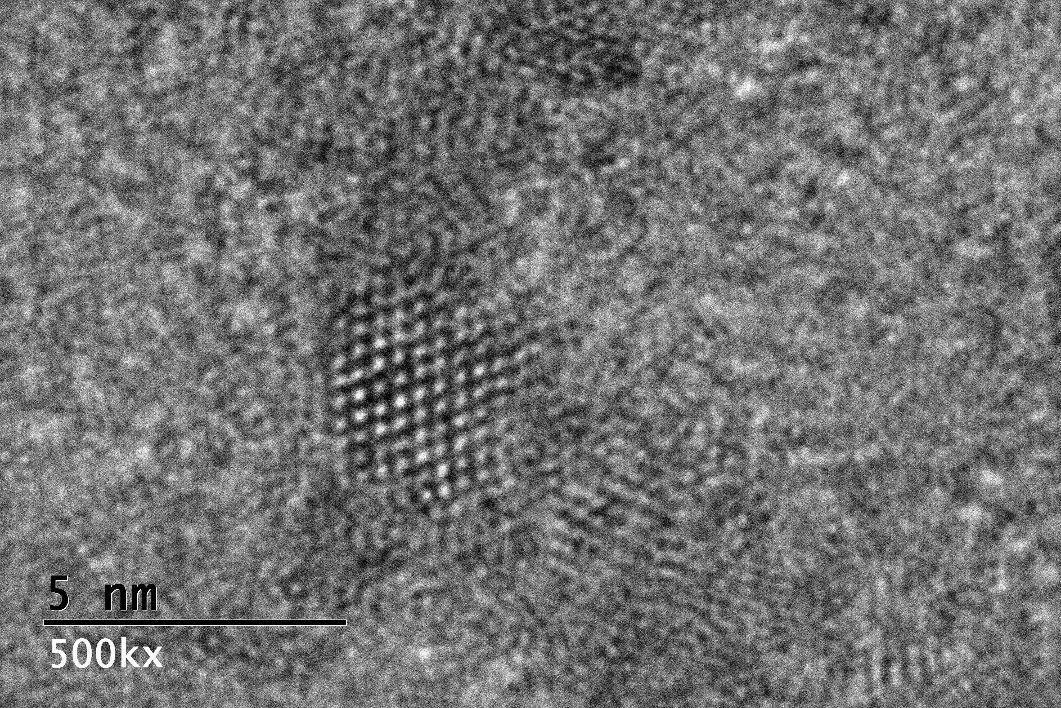

Supplement: Supplementary file 2 — Supplementary Material Details [file j_nanoph-2024-0529_suppl_002.zip › Supplemenatl Revised/TEM2.png]

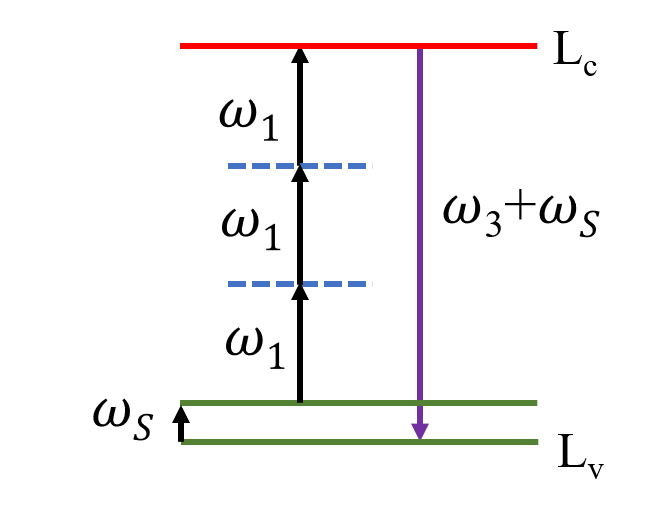

Supplement: Supplementary file 2 — Supplementary Material Details [file j_nanoph-2024-0529_suppl_002.zip › Supplemenatl Revised/HR.png]

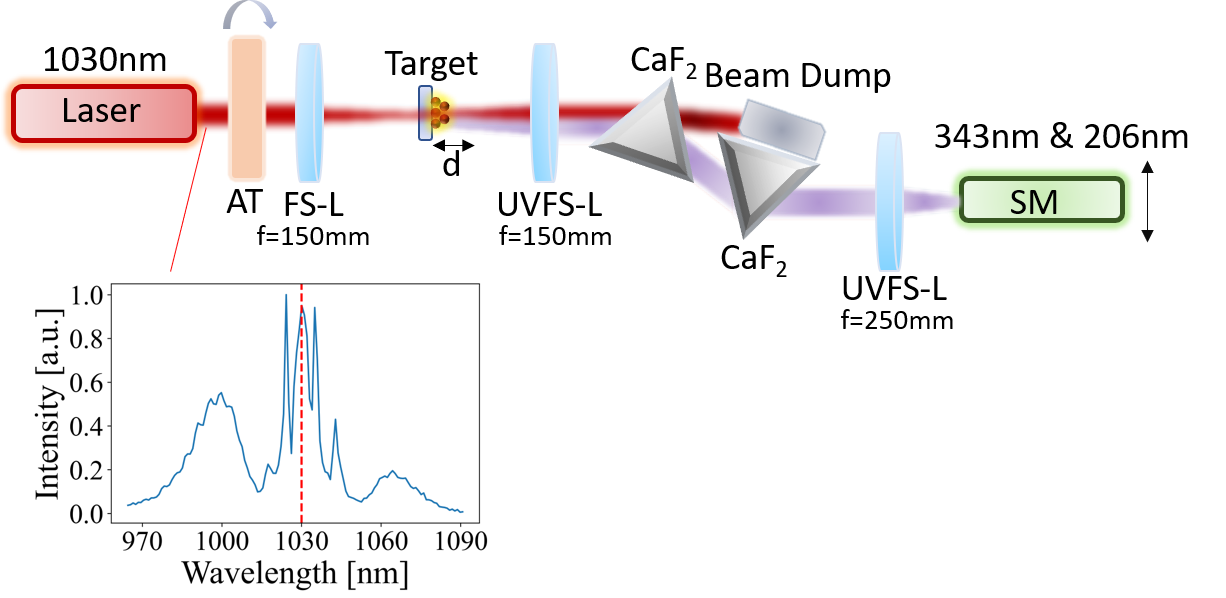

Supplement: Supplementary file 2 — Supplementary Material Details [file j_nanoph-2024-0529_suppl_002.zip › Supplemenatl Revised/AUFBAU_Tobi.png]
